# Boron-mediated sequential alkyne insertion and C–C coupling reactions affording extended *π*-conjugated molecules

**DOI:** 10.1038/ncomms12704

**Published:** 2016-09-01

**Authors:** Yoshiaki Shoji, Naoki Tanaka, Sho Muranaka, Naoki Shigeno, Haruka Sugiyama, Kumiko Takenouchi, Fatin Hajjaj, Takanori Fukushima

**Affiliations:** 1 Laboratory for Chemistry and Life Science, Institute of Innovative Research, Tokyo Institute of Technology, 4259 Nagatsuta, Midori-ku, Yokohama 226-8503, Japan

## Abstract

C–C bond coupling reactions illustrate the wealth of organic transformations, which are usually mediated by organotransition metal complexes. Here, we show that a borafluorene with a B–Cl moiety can mediate sequential alkyne insertion (1,2-carboboration) and deborylation/C*sp*^2^–C*sp*^2^ coupling reactions, leading to aromatic molecules. The first step, which affords a borepin derivative, proceeds very efficiently between the borafluorene and various alkynes by simply mixing these two components. The second step is triggered by a one-electron oxidation of the borepin derivative, which results in the formation of a phenanthrene framework. When an excess amount of oxidant is used in the second step, the phenanthrene derivatives can be further transformed *in situ* to afford dibenzo[*g*,*p*]chrysene derivatives. The results presented herein will substantially expand the understanding of main group chemistry and provide a powerful synthetic tool for the construction of a wide variety of extended *π*-conjugated systems.

## Abstract (web summary)

Coupling reactions to form C–C bonds typically require transition metals. Here the authors report that an organoboron compound can allow sequential alkyne insertion and unusual oxidative deborylation/*Csp^2^*–*Csp^2^* coupling, enabling the transformation of acetylenes into extended π-conjugated molecules.

## Graphical abstract

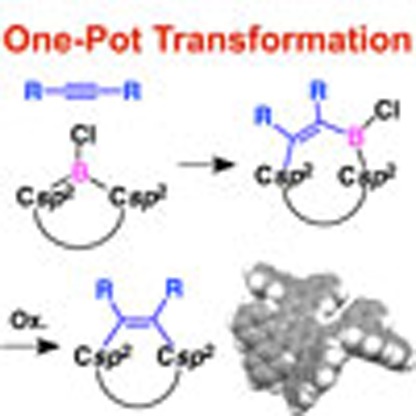

Recently, several studies have demonstrated that main group compounds can exhibit remarkable reactivity, which is typical of transition metals^1^. Pioneering works include the multiple complexation of CO with boron^2^, the reversible addition of ethylene to a distannyne^3^, and the activation of H_2_ with a carbene^4^ and frustrated Lewis pairs^5^,^6^ (Fig. 1a), all of which are characterized by the activation of small inert molecules with reactive main group centres. Meanwhile, in the context of organic transformations, many organotransition metal compounds allow the insertion of unsaturated compounds into their metal–carbon bonds, and some of the resulting complexes subsequently undergo elimination of the metal moiety to mediate cross-coupling reactions^7^,^8^ (Fig. 1b). This sequence of reactions still sets the chemistry of transition metals apart from that of main group elements. Here, we show that a three-coordinate borane with a B–Cl moiety, embedded in a *π*-conjugated five-membered ring, undergoes a sequential reaction that involves an alkyne insertion (1,2-carboboration) and a subsequent oxidative deborylation/C*sp*^2^–C*sp*^2^ coupling (Fig. 1c), which enables the conversion of alkynes into the corresponding benzannulated and cyclodehydrogenated products with extended *π*-conjugated frameworks.

Boron with strong Lewis acidity and low electronegativity is known to mediate C–C coupling reactions. Examples include 1,1- and 1,2-carboborations^9^,^10^,^11^,^12^,^13^,^14^,^15^,^16^,^17^,^18^, electrocyclizations^19^ and carbonylations^20^ of three-coordinate boranes, as well as the oxidative 1,2-migration of carbon substituents and the subsequent deborylation of four-coordinate borate anions^12^,^21^,^22^ (Supplementary Fig. 1). However, it has never been expected that one-electron oxidations of three-coordinate boranes result in C–C coupling reactions (Fig. 1c). Such reactions may seem unlikely, as they would require the elimination of the boron moiety from the radical cation state of the parent boranes under concomitant generation of a low-valent cationic boron species, which usually represents a very high-energy state^23^,^24^,^25^. In fact, the boron-mediated C*sp*^2^–C*sp*^2^ coupling reaction reported herein (Figs 1c and 2a) was observed serendipitously during our attempts to synthesize boron-substituted acetylenes through the metathesis reaction of 9-chloro-9-borafluorene (**1**) and bis(trimethylsilyl)acetylene (Fig. 2b). In contrast to our initial expectations, this reaction yielded boron-containing seven-membered-ring compound **5** almost quantitatively (Fig. 2b and Supplementary Fig. 3). Since the addition reaction of 9-borabicyclo[3.3.1]nonane to bis(trimethylsilyl)acetylene has previously been reported to be initiated by a hydroboration (Supplementary Fig. 2)^26^, we assumed that **5** was formed *via* the 1,2-carboboration of **1** and bis(trimethylsilyl)acetylene, followed by a 1,2-migration of the trimethylsilyl group. In that case, the 1,2-migration should be suppressed when bis(trimethylsilyl)acetylene is replaced with diphenylacetylene (**2a**). Indeed, when borafluorene **1** was treated with one equivalent of **2a**, the 1,2-carboboration occurred and yielded 5-chloro-6,7-diphenyl-5*H*-dibenzo[*b*,*d*]borepin (**3a**) exclusively (Fig. 2a). However, to our surprise, **3a** was immediately transformed into 9,10-diphenylphenanthrene (**4a**) on exposure to air. This absolutely unexpected observation prompted us to further explore the reactivity of these chloro-substituted borepins.

## Results

### Carboboration of diphenylacetylene with borafluorene

The 1,2-carboboration between **1** and **2a** in CH_2_Cl_2_ at 25 °C under an argon atmosphere yielded **3a** almost quantitatively, even though the reaction required 4 days for completion. When performed in 1,2-dichloroethane (DCE) at 80 °C, the reaction was completed within 12 h, and detectable byproducts were not observed. Thus, **3a** was isolated in 87% yield, simply by recrystallization of the reaction mixture under argon (see Supplementary Methods). The structure of **3a** was assigned unambiguously on the basis of ^1^H, ^13^C and ^11^B NMR as well as infrared spectroscopy, atmospheric pressure chemical ionization time-of-flight mass spectrometry and single-crystal X-ray diffraction analysis (Supplementary Fig. 4). So far, only a limited number of examples of such catalyst-free 1,2-carboboration reactions of alkynes and related unsaturated compounds have been reported; mostly for diarylchloroboranes^9^, boroles^10^,^11^,^12^,^13^,^14^,^15^ and borenium ions (three-coordinate boron cations)^16^,^17^. Borafluorene **1** can thus be considered as a readily available 1,2-carboboration reagent for acetylenes. In contrast, diphenylchloroborane, which is an acyclic C–B(Cl)–C system, did not react with diphenylacetylene **2a**, even in refluxing DCE. Hence, sterically less-hindered cyclic structures might facilitate the 1,2-carboboration with alkynes.

### Oxidative deborylation/C–C coupling of borepins

**Figure 2:**
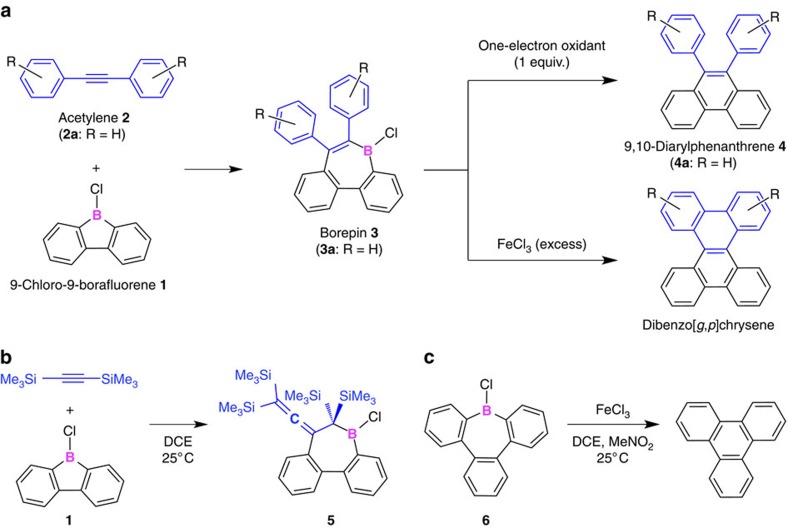
Boron-mediated C*sp*^2^–C*sp*^2^ coupling reactions. (**a**) 1,2-Carboboration of diarylacetylene **2** with borafluorene **1**, followed by a deborylation/C*sp*^2^–C*sp*^2^ coupling reaction in the presence of an oxidant. (**b**) Reaction between bis(trimethylsilyl)acetylene and **1**. (**c**) Oxidative deborylation/C*sp*^2^–C*sp*^2^ coupling reaction of **6**.

During the isolation of chloroborepin **3a**, we noticed that **3a** underwent a certain transformation on minimal contamination of the reaction mixture with air. The ^1^H NMR analysis identified 9,10-diphenylphenanthrene (**4a**) as the resulting product. Furthermore, the exposure of a DCE solution of **3a** at 25 °C to O_2_ immediately led to its transformation into **4a**, which could be isolated in 54% yield (Fig. 2a and Supplementary Table 3, entry 1). It seemed that the auto-oxidation induced the deborylation/C–C coupling of **3a** into **4a**, and therefore we further investigated the oxidation of **3a**. Remarkably, under very mild conditions using organic one-electron oxidants such as iodine, tris(4-bromophenyl)ammoniumyl hexachloroantimonate [(*p*-BrC_6_H_4_)_3_N^·+^][SbCl_6_^−^], or 2-azaadamantane-*N*-oxyl (AZADO), as well as FeCl_3_ or MnO_2_, **3a** could be converted efficiently into **4a** (Supplementary Table 3). The highest isolated yield of **4a** (94%) was obtained when FeCl_3_ or MnO_2_ were used (Supplementary Table 3, entries 4 and 5). The oxidation reactions using [(*p*-BrC_6_H_4_)_3_N^·+^][SbCl_6_^−^] and FeCl_3_ (Supplementary Table 3, entries 2 and 4) indicated that the conversion from **3a** to **4a** requires essentially only one equivalent of oxidant. The deborylation/C–C coupling reaction of **3a** into **4a** can also be achieved by electrochemical oxidation, which involves an electron transfer at an electrode surface. Cyclic voltammetry of **3a** in *o*-dichlorobenzene (ODCB), which contained tetrabutylammonium hexafluorophosphate [Bu_4_N^+^][PF_6_^−^] as the supporting electrolyte, showed an irreversible oxidation wave at 1.05 V (versus ferrocene/ferrocenium) during the first anodic scan (Supplementary Fig. 9). After the second cycle, the height of the oxidation wave of **3a** markedly decreased and, instead, a reversible oxidation wave with a half wave potential of 1.18 V appeared, which perfectly matched the first oxidation potential of **4a** under identical electrolysis conditions (Supplementary Fig. 10). The ^1^H NMR analysis of a mixture of **3a** and [Bu_4_N^+^][PF_6_^−^] in ODCB-*d*_4_ confirmed that the boron atom of **3a** is free-from coordination by the supporting electrolyte used in the electrochemical measurements.

Similar to **3a**, fully benzannulated chloroborepin **6** also underwent this deborylation/C–C coupling reaction (Fig. 2c). When **6** was treated with an equimolar amount of FeCl_3_ at 25 °C, triphenylene was formed quantitatively (see Supplementary Methods). This result, together with those observed for **3a**, clearly indicates that one-electron oxidation of the chloroborepins **3a** and **6** results in the spontaneous release of their boron functionality to cause the C*sp*^2^–C*sp*^2^ coupling. When taking the material balance of the deborylation/C–C coupling reaction into account, a low-valent cationic boron species such as [B–Cl]^·+^ should thus be generated from the borepin radical cations **3a**^·+^ and **6**^·+^. At present, we presume that solvent molecules and/or anionic species from the oxidants could interact with the boron centre of the radical cations to assist the formal elimination of [B–Cl]^·+^. Despite numerous efforts, we have been unable to isolate any products arising from the eliminated boron moiety.

### Substrate scope

So far, several reactive main group compounds have been reported to behave like transition metal compounds in the activation of small inert molecules^1^,^2^,^3^,^4^,^5^,^6^. The sequential 1,2-carboboration and oxidative deborylation/C–C coupling reactions between borafluorene **1** and alkynes are useful for the construction of a diverse range of aromatic systems (Table 1) in a one-pot procedure (see the ‘Methods' section and Supplementary Methods). Typically, under an argon atmosphere, internal alkyne **2** was allowed to react with borafluorene **1** (1.1 equivalents) in DCE at 80 °C for 12 h, and then a nitromethane solution containing FeCl_3_ (1.1 equivalents relative to **2**) was added to the resulting mixture. The ^1^H NMR analysis confirmed that the catalyst-free 1,2-carboboration using borafluorene **1** proceeded very efficiently for all internal alkynes used, and afforded the corresponding borepins in excellent yields (**3b**–**3j**: 83–99%; Table 1). After oxidation, 9,10-diarylphenanthrenes with 4-methyl and 4-methoxy groups were isolated in good yield (**4b**: 85%, **4c**: 82%; Table 1). Similarly, 9,10-diarylphenanthrenes with halogen functionalities were obtained in high yield (**4d**: 82%, **4e**: 80%; Table 1). The use of diarylacetylenes with strongly electron-withdrawing 4-trifluoromethyl or polar Lewis-basic 4-methoxycarbonyl groups resulted in lower isolated yields of the product (**4f**: 67%, **4g**: 48%; Table 1). Moreover, di(thiophene-1-yl)acetylene could be converted into 9,10-bis(thiophene-1-yl)phenanthrene (**4h**: 73%; Table 1). Interestingly, internal alkynes with aryl or alkyl substituents (**4i**: 64%; Table 1), and even with fully aliphatic substituents (**4j**: 71%; Table 1) were also susceptible to the sequential 1,2-carboboration and oxidative deborylation/C–C coupling reactions with **1**. A terminal alkyne could also be converted into the corresponding phenanthrene derivative (**4k**), albeit in moderate yield (34%; Table 1). Notably, stilbene was unreactive in the presence of **1**, even in refluxing ODCB, indicating that the 1,2-carboboration is chemoselective for alkynes.

The one-pot, sequential 1,2-carboboration and oxidative deborylation/C–C coupling reactions are moreover applicable to substrates with multiple acetylene moieties (Table 2). For example, 1,4-bis(10-phenylphenanthren-9-yl)benzene (**8**) was obtained in 88% isolated yield from **1** and 1,4-bis(phenylethynyl)benzene (**7**) when 2.2 equivalents of FeCl_3_ were used. When a large excess of FeCl_3_ (for example, 30 equivalents relative to **7**) was used, a Scholl-type cyclization^27^ of as-formed **8** occurred simultaneously to generate hexabenzo[*a*,*c*,*f*,*j*,*m*,*o*]picene (**9**; 89%), which features a [5]helicene structure. Similarly, the treatment of a reaction mixture of **1** and 1,3-bis(phenylethynyl)benzene (**10**) with 2.2 or 30 equivalents of FeCl_3_ furnished 1,3-bis(10-phenylphenanthren-9-yl)benzene (**11**) or tetrabenzo[*a*,*c*,*f*,*k*]phenanthro[9,10-*m*]tetraphene (**12**) in 89 and 85% yield, respectively. Remarkably, borafluorene **1** also reacts with sterically demanding internal alkynes, which then undergo the oxidative deborylation/C–C coupling. For example, 1,3,5-tris(phenylethynyl)benzene (**13**) was converted into 1,3,5-tris(10-phenylphenanthren-9-yl)benzene (**14**) in moderate yield (35%), and **16**, with two anthracene moieties in close proximity to each other, could be synthesized from 1,2-di(anthracen-9-yl)ethyne (**15**). The successful preparation of single crystals of **9**, **12** and **14** allowed the determination of their molecular structures by X-ray diffraction analysis (Supplementary Figs 5–7). Compounds **9** and **12**, containing multiple dibenzo[*g*,*p*]chrysene^28^ moieties, adopt a highly twisted structure and feature a curved *π*-conjugated skeleton (Supplementary Figs 5 and 6). Using a procedure similar to that for bifunctional acetylenes, 1,4-diphenylbuta-1,3-diyne (**1**7) could be benzannulated with **1** to afford 10,10′-diphenyl-9,9′-biphenanthrene (**18**)^29^ in 80% isolated yield (Table 2). This product was obtained as a racemic mixture of *P*- and *M*-helical chiral enantiomers, which did not exhibit helix inversion at 25 °C, most likely on account of the intrinsic steric congestion. The enantiomers could therefore be separated using chiral high-performance liquid chromatography (Supplementary Fig. 11). The fact that even sterically demanding substrates such as **13**, **15** and **17** are easily transformed into the corresponding benzannulated products demonstrates the great potential and synthetic utility of the sequential 1,2-carboboration and oxidative deborylation/C–C coupling for the construction of extended *π*-conjugated systems with complex three-dimensional structures.

To further examine the versatility of the present benzannulation of acetylenes, we converted *meta*-phenylene ethynylene oligomers **19** and **21** into highly extended *π*-conjugated systems (Table 2). In this context, hexamer **21** is especially interesting, as it gives rise to a large helical molecule, which may potentially possess chiroptical properties. Oligomers **19** and **21** were successfully transformed into the desired products **20** and **22**, respectively, on reaction with **1** and subsequent oxidation with an excess of FeCl_3_ (Table 2; see Supplementary Methods). Powder X-ray diffraction analysis of the isolated products revealed that **20** is crystalline, while **22** is amorphous. Compound **22** displayed very broad signals in the ^1^H NMR spectrum (Supplementary Fig. 15), most likely due to rich conformational isomerism. Fourier-transform ion cyclotron resonance mass spectra clearly showed the ion peaks for **20** (calculated for C_86_H_46_ [M]^+^: *m*/*z*=1,078.3594; found: 1,078.3594) and **22** (calculated for C_126_H_66_ [M]^+^: *m*/*z*=1,578.5159; found: 1,578.5147; Supplementary Figs 14 and 17). Moreover, the single-crystal X-ray crystallographic analysis of **20** revealed an extended *π*-conjugated skeleton with a heavily twisted geometry (Supplementary Fig. 8).

## Discussion

We have demonstrated an unprecedented reaction of organoboron compounds, which allows the facile transformation of acetylenes into non-planar, curved three-dimensional *π*-conjugated molecules. Such curved and/or twisted *π*-conjugated systems have recently received considerable attention, as they can exhibit dynamic structural changes^30^,^31^,^32^, chiroptical properties^33^,^34^,^35^ and characteristic assembling behaviour, which may be beneficial for applications in organic electronics^28^,^36^. Besides the synthetic utility, the sequential nature of this protocol, featuring the insertion of an alkyne (1,2-carboboration) into a B–C bond, followed by an oxidative deborylation/C–C coupling reaction, sets it substantially apart from hitherto known reactions mediated by main group elements. Although the boron species released from the chloroborepin radical cations has not yet been identified, this unexpected reactivity will most likely spark new interest in the scientific community to promote further experimental and theoretical investigations in this area.

## Methods

### General

Air- and/or moisture-sensitive compounds were handled either in an argon-filled glove box or by applying standard Schlenk-line techniques. Anhydrous CH_2_Cl_2_ and hexane were dried by successive passage through an activated alumina column and a Q-5 column (Nikko Hansen & Co, Ltd). DCE, nitromethane (MeNO_2_), ODCB, CDCl_3_ and ODCB-*d*_4_ were dried over CaH_2_ and distilled immediately before use.

### General procedure for the on-pot synthesis of phenanthrenes 4a–4k

Under argon, a dry DCE solution (2.0 ml) of a mixture of **1** (109 mg, 0.55 mmol) and the corresponding acetylene (0.50 mmol) was stirred for 12 h at 80 °C and then allowed to cool to 25 °C (note: at this stage, the yield of the corresponding borepin derivative (**3a**–**3k**) was calculated on the basis of the ^1^H NMR spectroscopic analysis of the reaction mixture using hexamethylbenzene as the internal standard). After adding a dry MeNO_2_ solution (1.0 ml) of FeCl_3_ (81 mg, 0.50 mmol), the resulting mixture was stirred for 30 min at 25 °C and then poured into MeOH (150 ml). The precipitate thus formed was collected by filtration, dissolved in CH_2_Cl_2_, passed through a plug of Florisil, and evaporated to dryness under reduced pressure to afford the corresponding phenanthrene (**4a**–**4k**) (note: in some cases, an additional amount of the phenanthrene product could be isolated from the methanol filtrate by extraction with CH_2_Cl_2_, followed by SEC purification with CHCl_3_ as the eluent).

### Data availability

Crystal data of **3a**, **5**, **9**, **12**, **14** and **20** are available from the Cambridge Crystallographic Data Centre under reference numbers CCDC 1450400–1450405 via www.ccdc.cam.ac.uk/data_request/cif. For the analytical data of the compounds in this article including the NMR spectra, mass spectra, crystal data, a HPLC profile and cyclic voltammograms, see Supplementary Methods, Supplementary Figs 3–17 and Supplementary Tables 1–2. The authors declare that the other data supporting the findings of this study are available on request.

## Additional information

**How to cite this article:** Shoji, Y. *et al* Boron-mediated sequential alkyne insertion and C–C coupling reactions affording extended *π*-conjugated molecules. *Nat. Commun.* 7:12704 doi: 10.1038/ncomms12704 (2016).

## Supplementary Material

Supplementary Information Supplementary Figures 1-17, Supplementary Tables 1-3, Supplementary Methods and Supplementary References

## Figures and Tables

**Figure 1:**
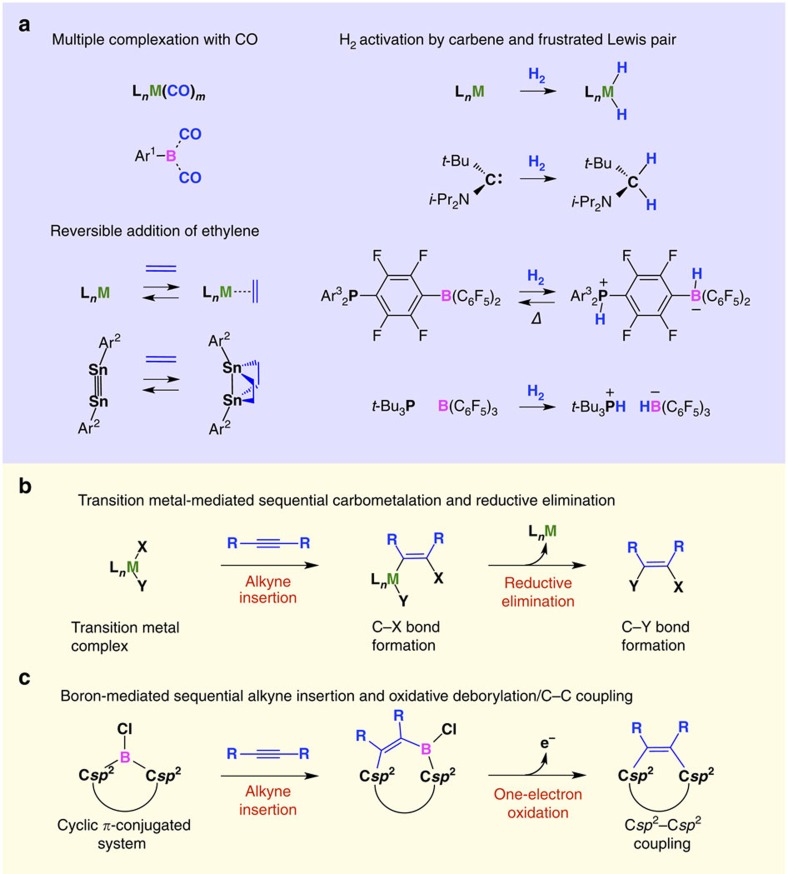
Examples of transition metal-like reactivity of main group compounds and C-C coupling reactions mediated by boron and transition metals. (**a**) Reported examples of transition metal-like reactivity of main group compounds. Ar^1^=2,6-bis(2,4,6-triisopropylphenyl)phenyl; Ar^2^=2,6-bis(2,6-triisopropylphenyl)phenyl; Ar^3^=2,4,6-trimethylphenyl. (**b**) Sequential alkyne insertion and reductive elimination reactions mediated by organotransition metal complexes. (**c**) Sequential alkyne insertion (1,2-carboboration) and oxidative deborylation/C*sp*^2^–C*sp*^2^ coupling reaction from C–B(Cl)–C bonds embedded in cyclic *π*-conjugated systems. The first step, which affords a borepin derivative, proceeds very efficiently between the borafluorene and various alkynes by simply mixing the two components. The second step is triggered by a one-electron oxidation of the borepin derivative. This sequential reaction is chemoselective for alkynes and can be executed in a one-pot manner. L, ligand; M, transition metal; X and Y, non-metal elements.

**Table 1:**
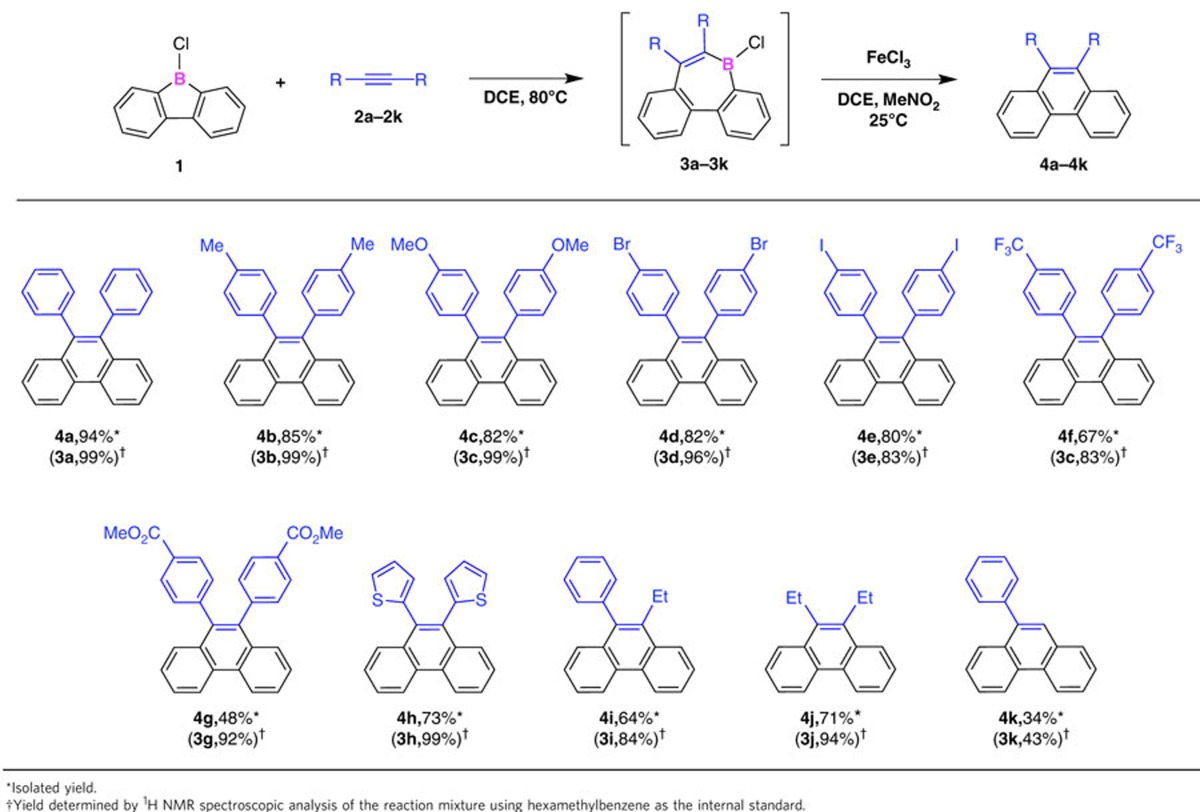
Substrate scope of the one-pot reaction between 1 and various types of acetylenes.

**Table 2:**
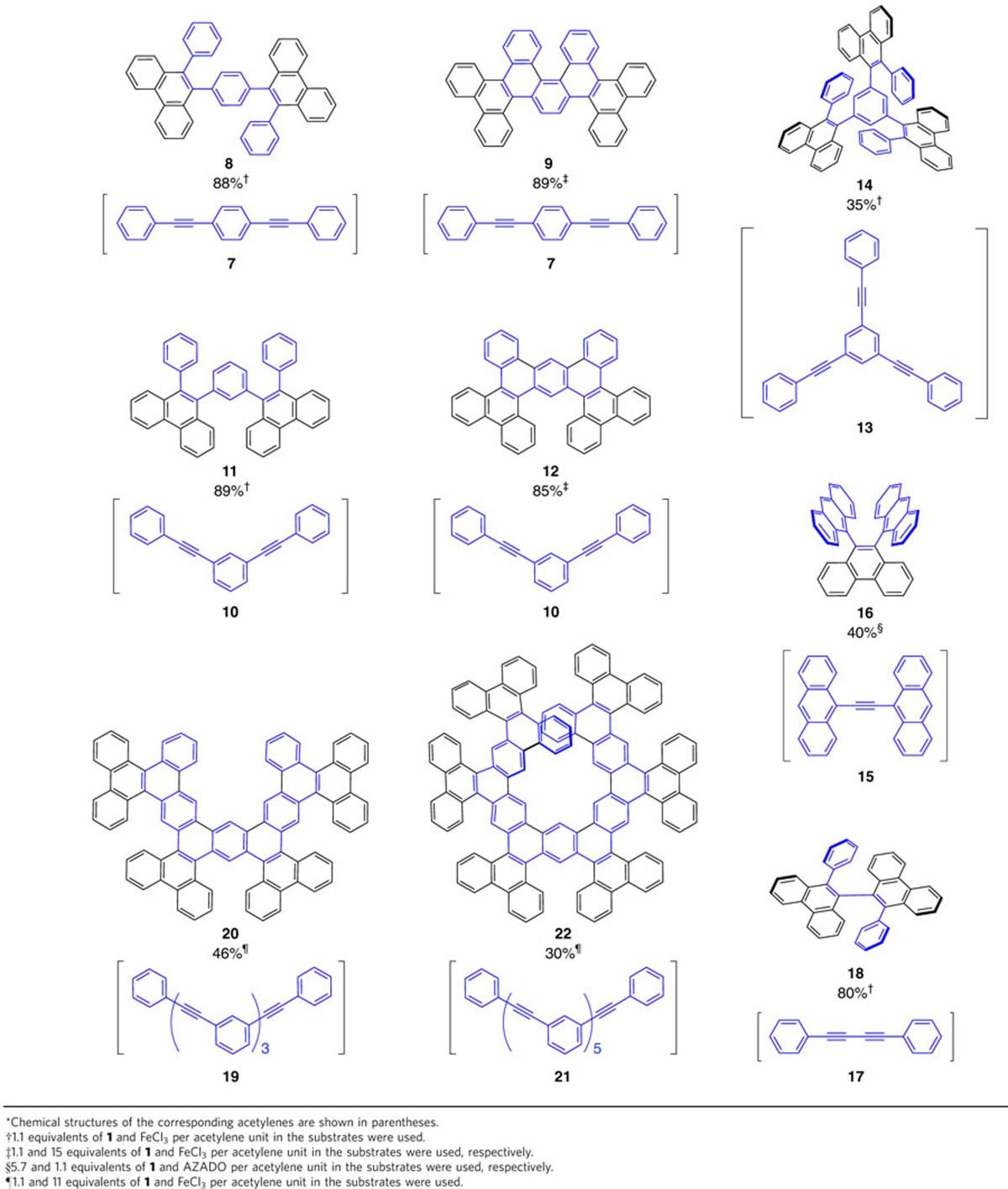
Benzannulated and cyclodehydrogenated products obtained from the one-pot reaction between 1 and various types of acetylenes.^*^
