# Supplementary material for: Boron-mediated sequential alkyne insertion and C–C coupling reactions affording extended π-conjugated molecules
Source: Nat Commun. 2016 Sep 1;7:12704. doi: 10.1038/ncomms12704 (PMC5025816; doi:10.1038/ncomms12704)
Supplement: Supplementary Information — Supplementary Figures 1-17, Supplementary Tables 1-3, Supplementary Methods and Supplementary References [file ncomms12704-s1.pdf]

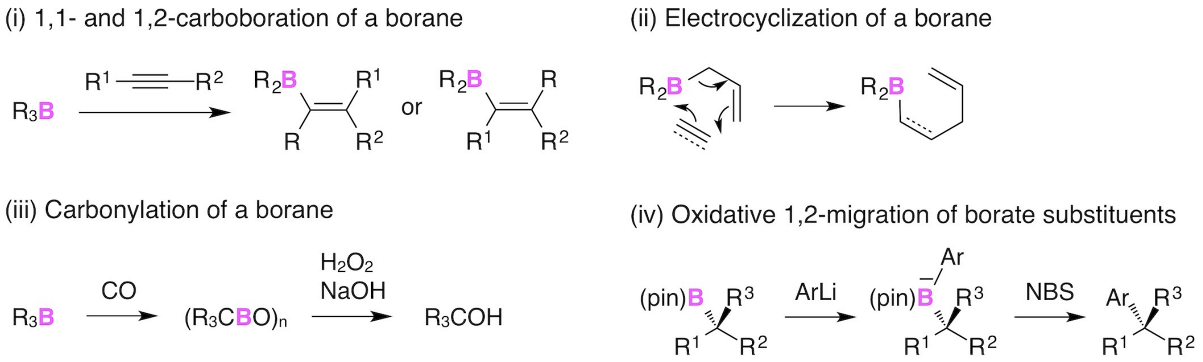

Supplementary Figure 1. Examples of C–C coupling reactions associated with a bond rearrangement at a single boron centre.

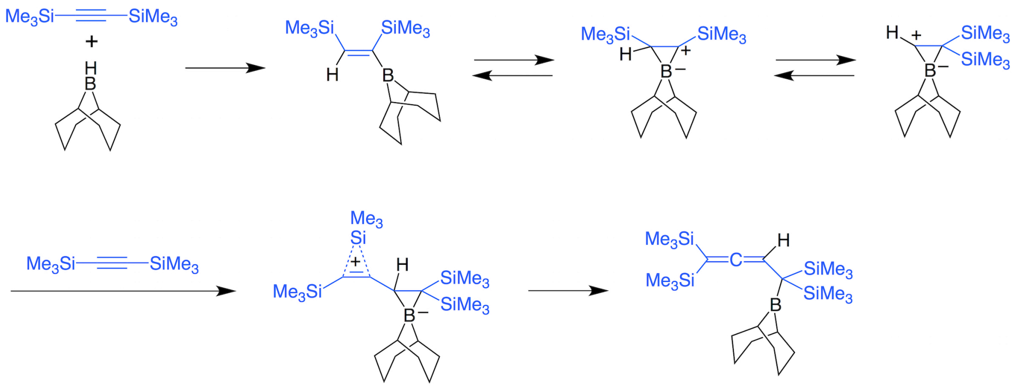

Supplementary Figure 2. Proposed mechanism for the reaction of 9-borabicyclo[3.3.1]nonane with two equivalents of bis(trimethylsilyl)acetylene (see Supplementary Reference 1).

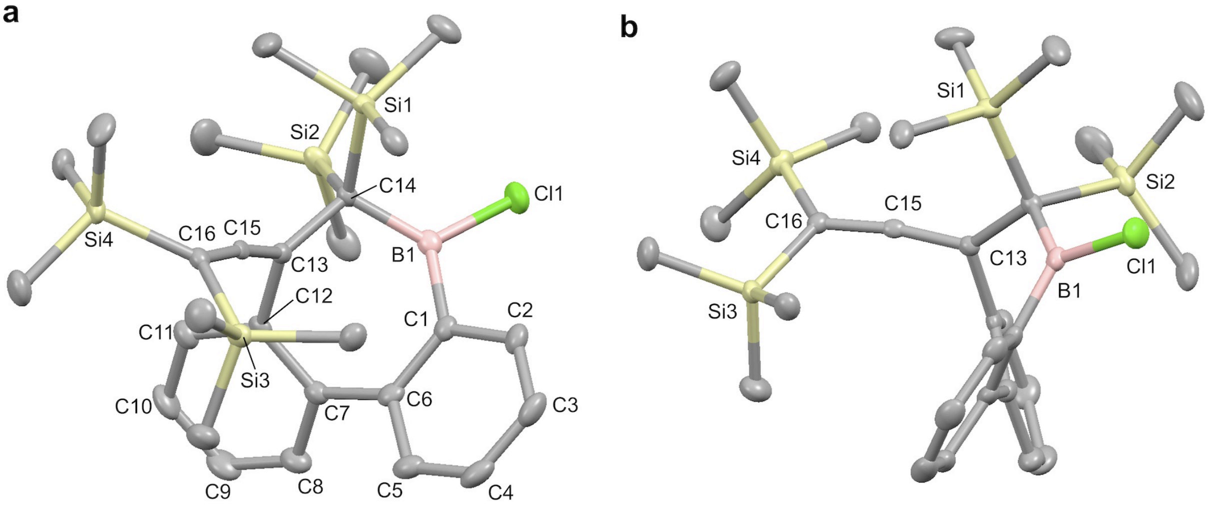

Supplementary Figure 3. Molecular structure of **5** with atomic displacement parameters set at 50% probability. **a**, top view. **b**, side view. Hydrogen atoms are omitted for clarity. Color code: boron = pink, carbon = gray, chlorine = green, silicon = yellow. Selected bond lengths (Å) and angles (°): Cl1–B1 = 1.8007(17), C1–B1 = 1.562(2), C1–C2 = 1.407(2), C1–C6 = 1.413(2), C2–C3 = 1.387(2), C3–C4 = 1.385(2), C4–C5 = 1.384(2), C5–C6 = 1.403(2), C6–C7 = 1.485(2), C7–C8 = 1.404(2), C7–C12 = 1.4085(19), C8–C9 = 1.386(2), C9–C10 = 1.387(2), C10–C11 = 1.389(2), C11–C12 = 1.396(2), C12–C13 = 1.5090(19), C13–C14 = 1.5496(19), C13–C15 = 1.3163(18), C14–B1 = 1.554(2), C14–Si1 = 1.9395(14), C14–Si2 = 1.9425(14), C15–C16 = 1.3114(19), C16–Si3 = 1.8940(14), C16–Si4 = 1.8829(15), C13–C15–C16 = 171.03(14).

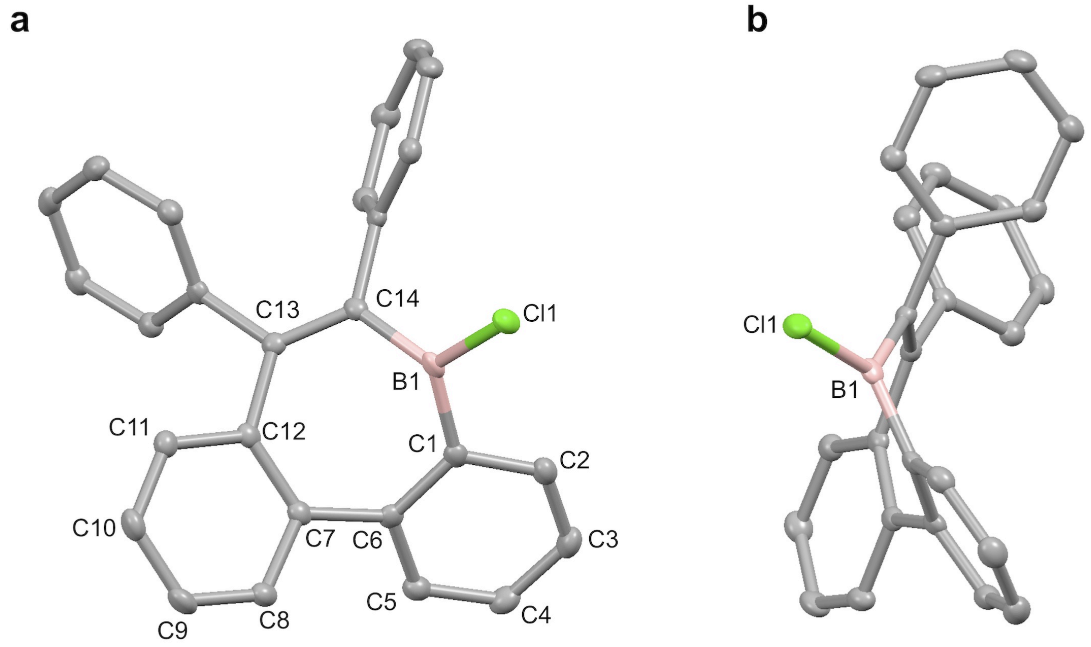

Supplementary Figure 4. Molecular structure of **3a** with atomic displacement parameters set at 50% probability. **a**, top view. **b**, side view. Hydrogen atoms are omitted for clarity. Color code: boron = pink, carbon = gray, chlorine = green. Selected bond lengths (Å) and angles (°): Cl1–B1 = 1.7747(15), C1–B1 = 1.5564(19), C1–C2 = 1.4040(19), C1–C6 = 1.4130(18), C2–C3 = 1.3876(18), C3–C4 = 1.383(2), C4–C5 = 1.3896(19), C5–C6 = 1.4035(18), C6–C7 = 1.4854(18), C7–C8 = 1.4077(17), C7–C12 = 1.4177(17), C8–C9 = 1.3787(19), C9–C10 = 1.388(2), C10–C11 = 1.385(2), C11–C12 = 1.4062(18), C12–C13 = 1.4982(17), C13–C14 = 1.3570(17), C14–B1 = 1.5564(19), C1–C6–C7–C12 = 43.14(18), C12–C13–C14–B1 = 5.23(19), C13–C14–B1–C1 = 52.40(17), C14–B1–C1–C6 = –41.45(18).

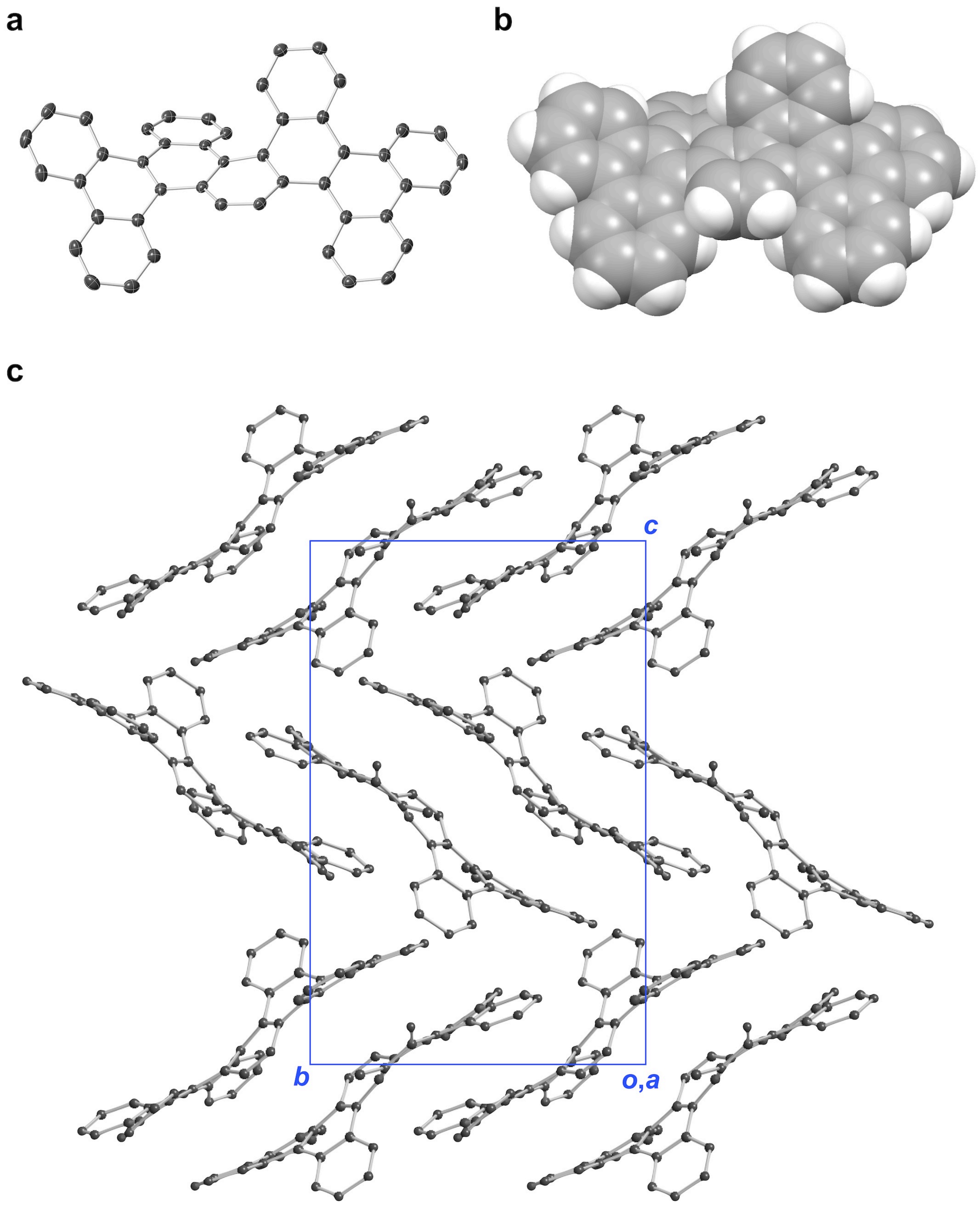

Supplementary Figure 5. (a) Molecular structure with atomic displacement parameters set at 50% probability, (b) space-filling model, and (c) packing diagram (*a* projection) of **9**. Color code: hydrogen = white, carbon = gray.

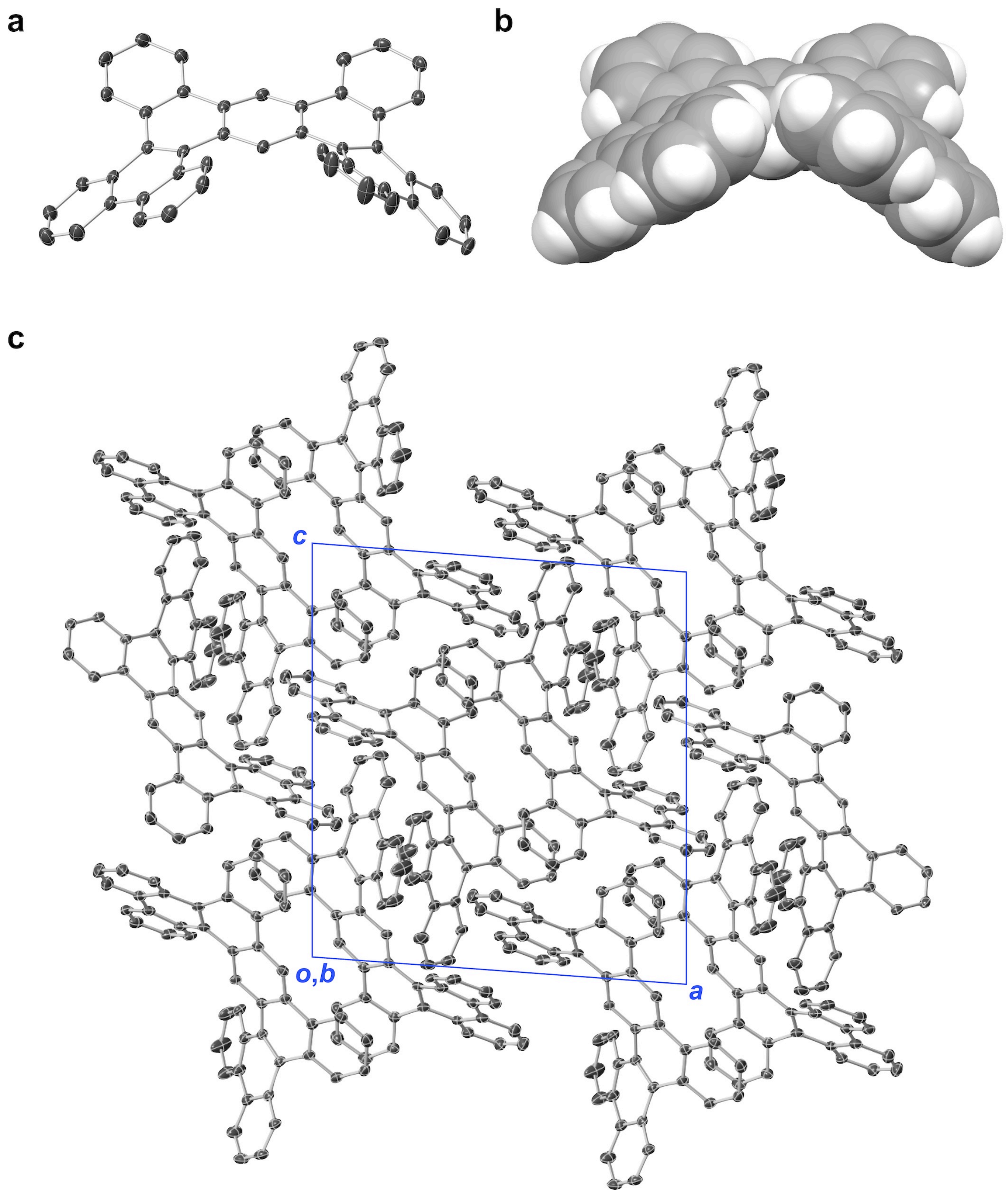

Supplementary Figure 6. (a) Molecular structure with atomic displacement parameters set at 50% probability, (b) space-filling model, and (c) packing diagram (*b* projection) of **12**. Co-crystallized toluene molecules are omitted for clarity. Color code: hydrogen = white, carbon = gray.

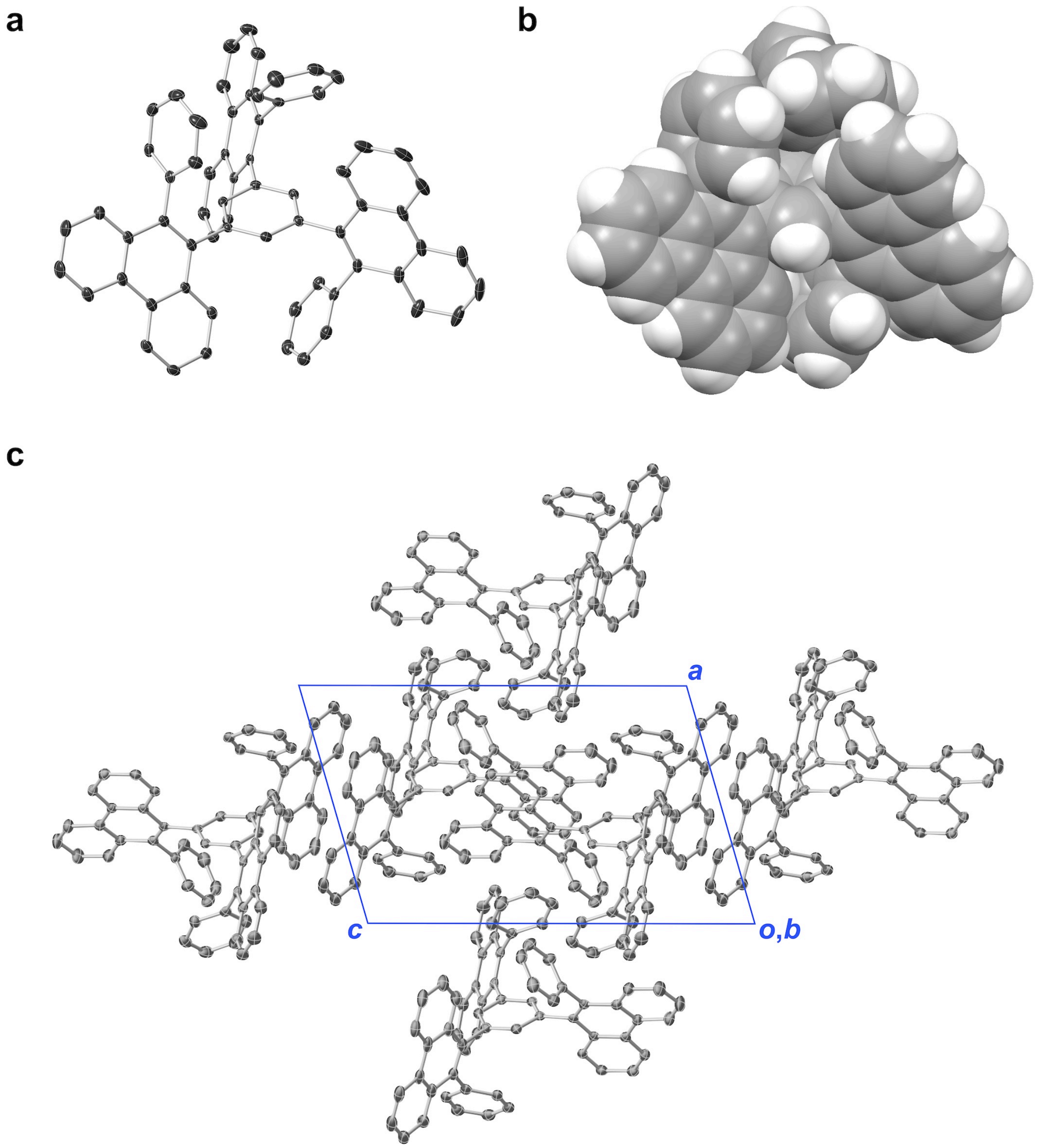

Supplementary Figure 7. (a) Molecular structure with atomic displacement parameters set at 50% probability, (b) space-filling model, and (c) packing diagram (*b* projection) of **14**. Co-crystallized benzene molecules are omitted for clarity. Color code: hydrogen = white, carbon = gray.

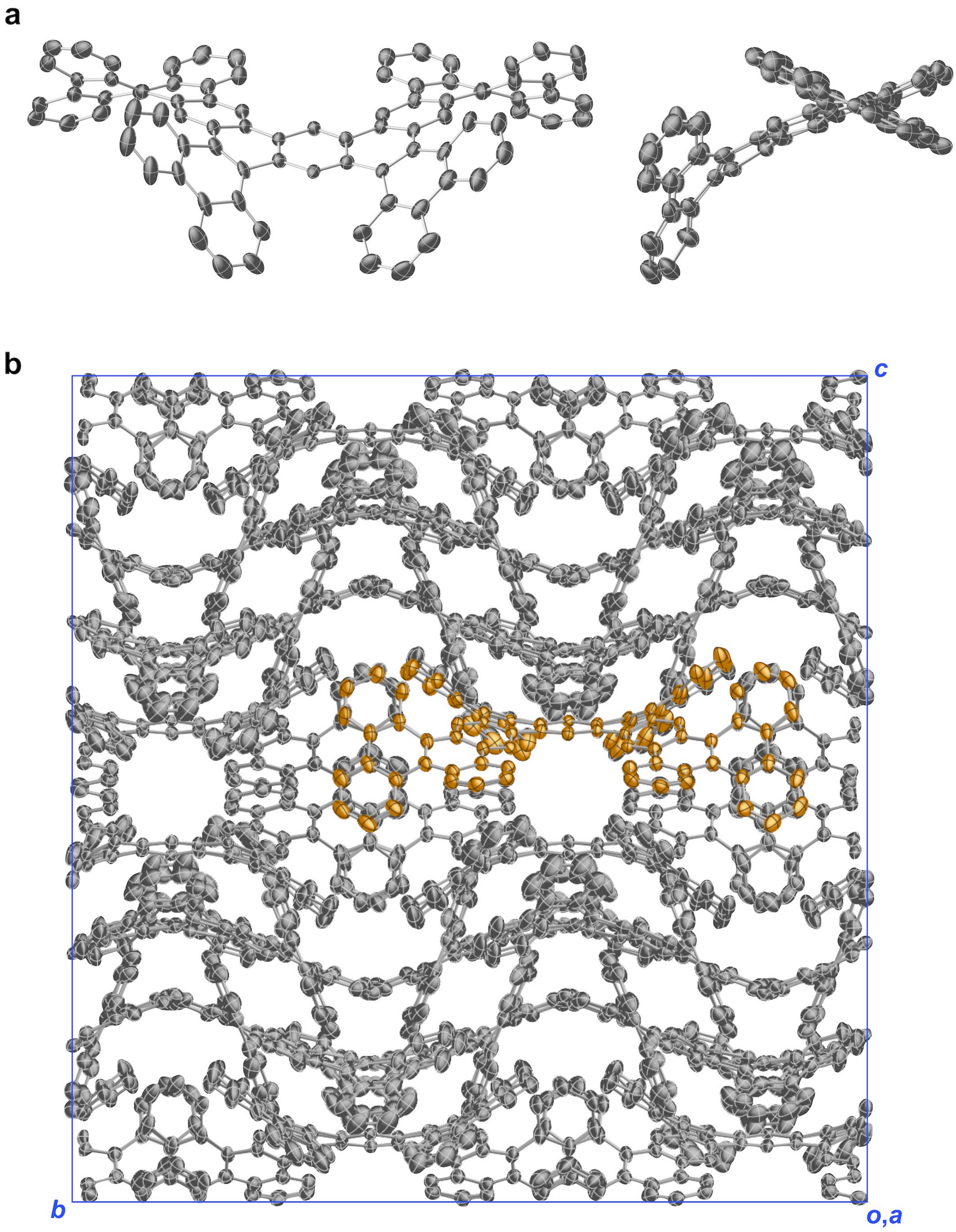

Supplementary Figure 8. (a) Molecular structure of one of the four similar independent molecules of **20** (left: top view, right: side view) with atomic displacement parameters set at 50% probability and (b) packing diagram (*a* projection). For clarity, one of the four molecules of **20** is colored in orange, and hydrogen atoms and co-crystallized CS_2 molecules are omitted. Color code: carbon = gray.

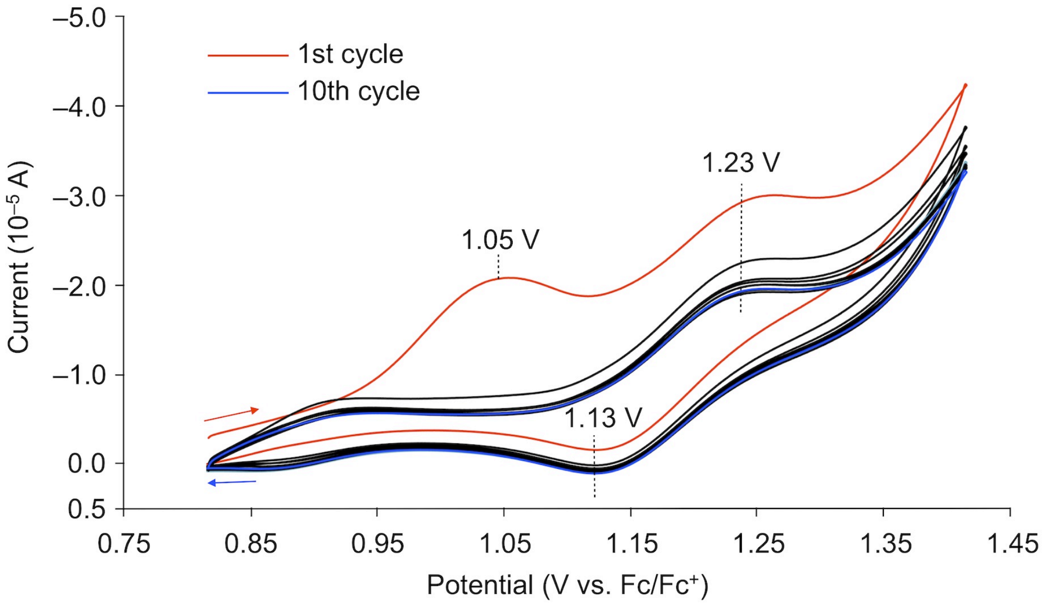

Supplementary Figure 9. Cyclic voltammograms of borepin **3a** (1.0×10^{-4} M) in ODCB, containing $[Bu_4N^+][PF_6^-]$ (0.1 M) as the supporting electrolyte. Working electrode: glassy carbon; counter electrode: Pt wire; pseudo-reference electrode: Ag wire; scan rate: 50 mV/s.

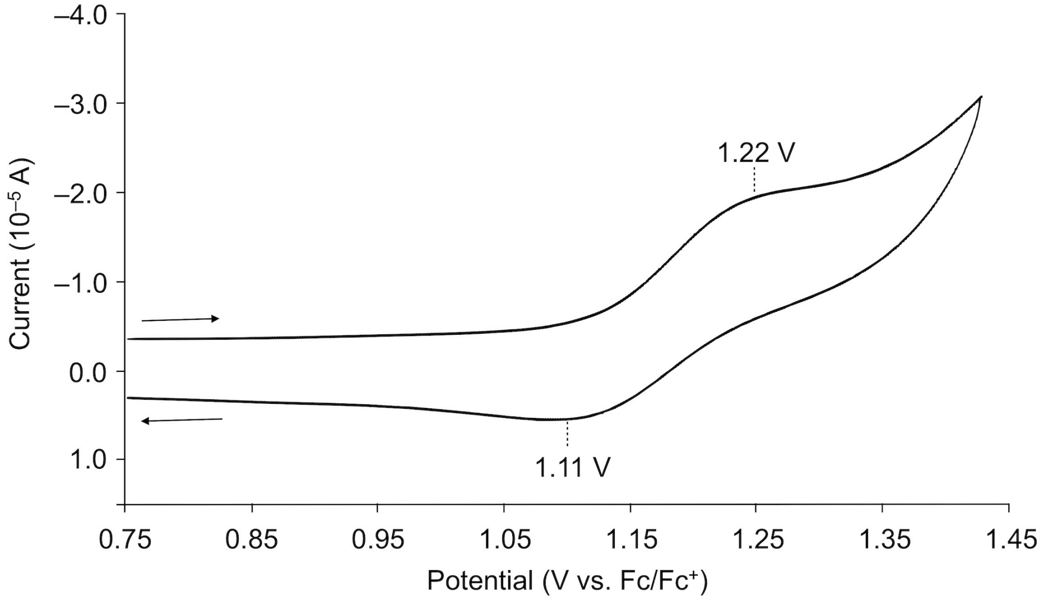

Supplementary Figure. 10. Cyclic voltammogram of 9,10-diphenylphenanthrene **4a** (1.0×10^{-4} M) in ODCB, containing $[Bu_4N^+][PF_6^-]$ (0.1 M) as the supporting electrolyte. Working electrode: glassy carbon; counter electrode: Pt wire; pseudo-reference electrode: Ag wire; scan rate: 50 mV/s.

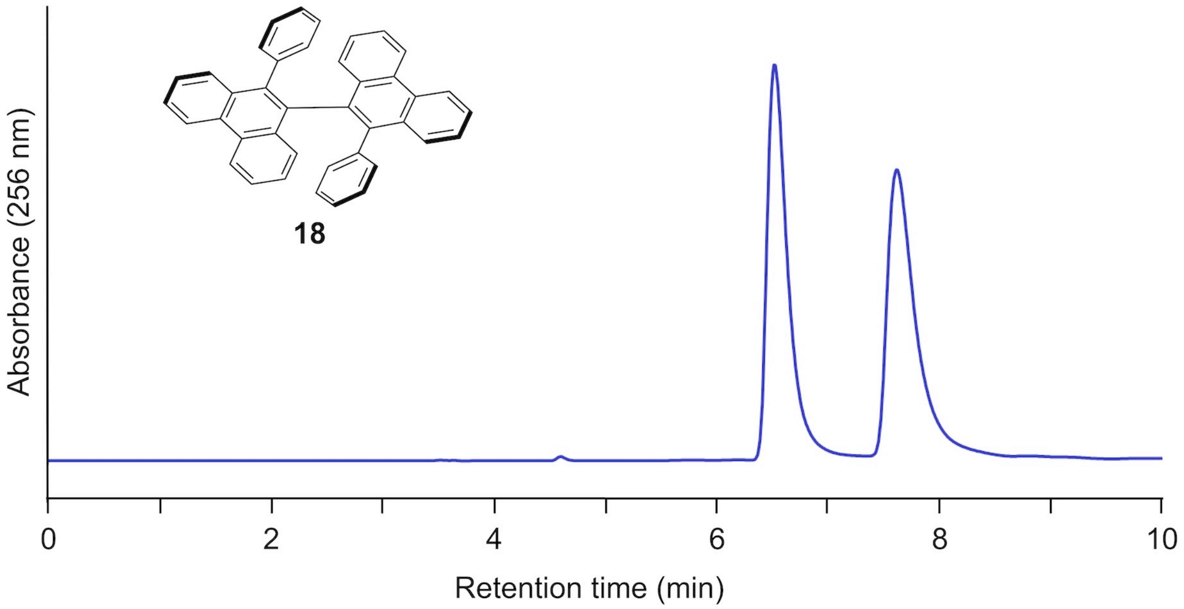

Supplementary Figure 11. Chiral HPLC profile of **18**, obtained at 25 °C from an Amylose-SA column (4.6 mm × 250 mm) with hexane/CH_2Cl_2 (v/v = 95/5) as the eluent at a flow rate of 1.0 mL/min (detection wavelength = 256 nm).

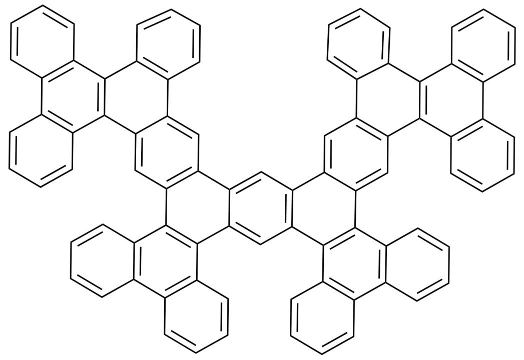

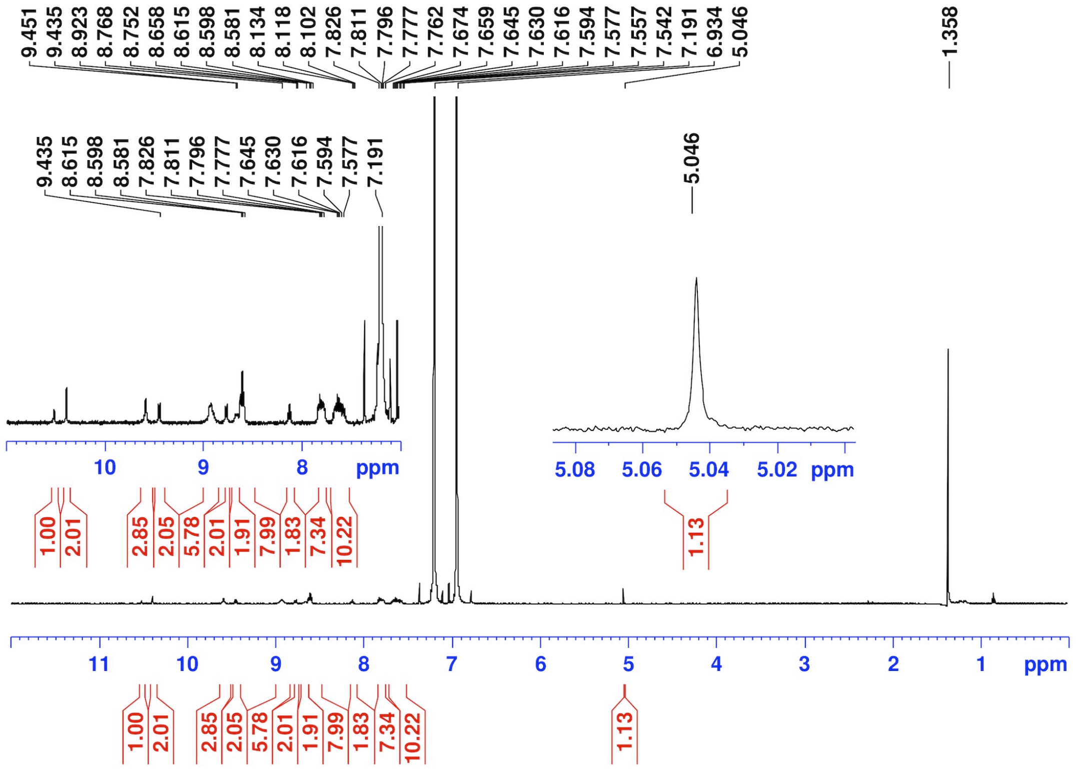

Supplementary Figure 12. ^{1}H NMR spectrum (500 MHz) of **20** in ODCB-d_4 at 25 °C.

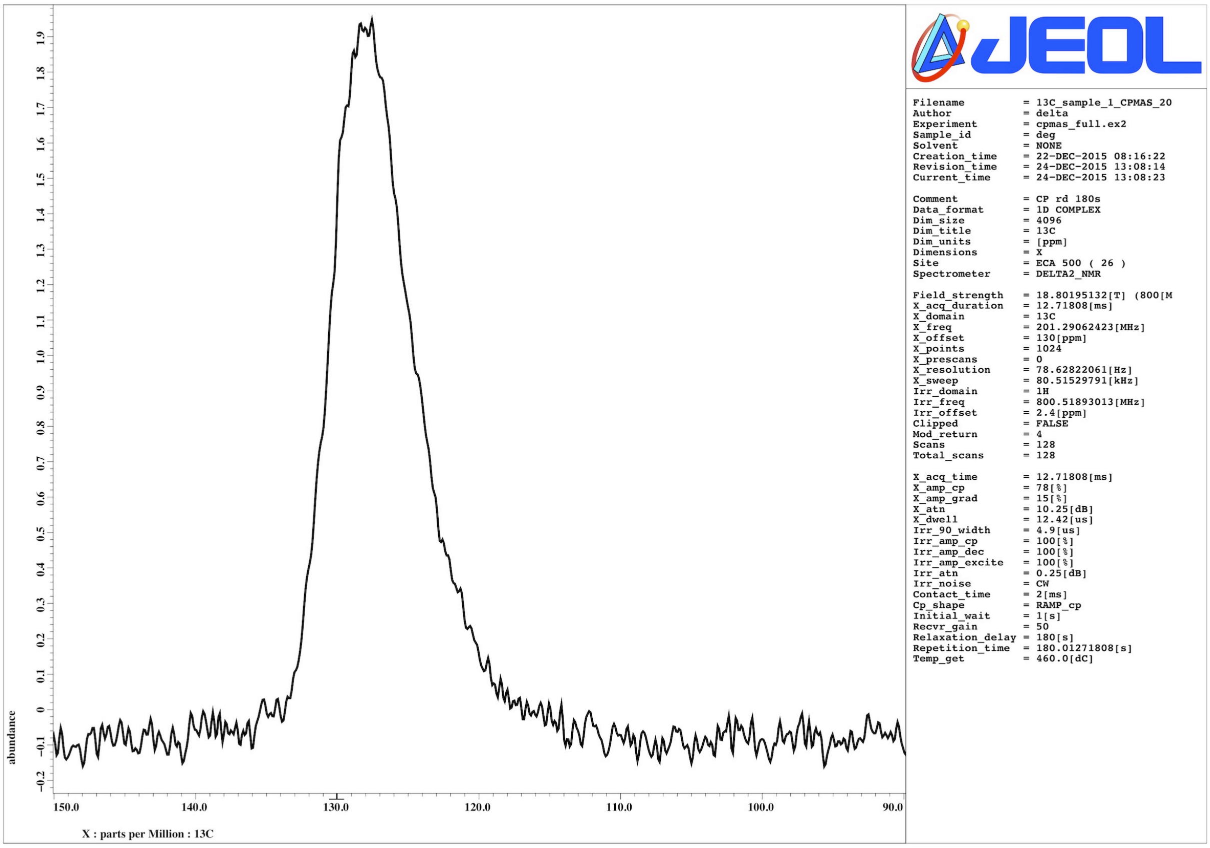

Supplementary Figure 13. CP/MAS ^{13}C NMR spectra of **20** (relaxation delay = 180 s) at 25 °C.

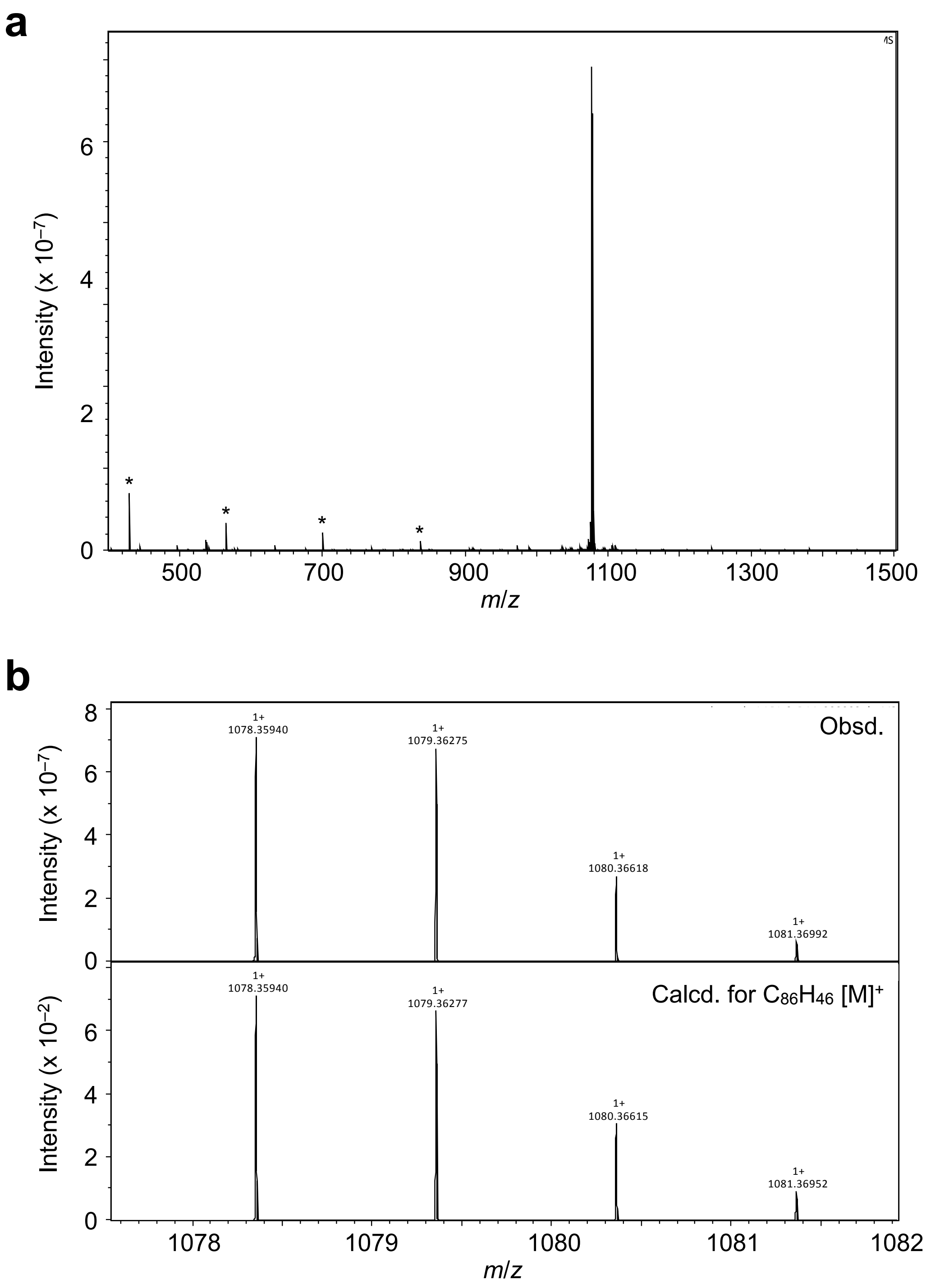

Supplementary Figure 14. FT-ICR MS spectrum of **20** (a) and magnifications for the area m/z = 1077–1082 (b). Peaks associated with asterisks arise from sodium trifluoroacetate as the internal standard.

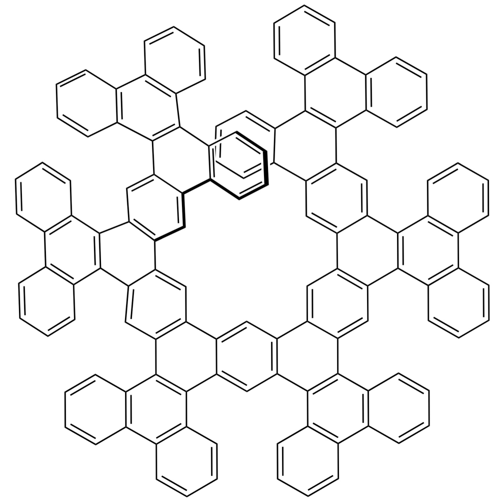

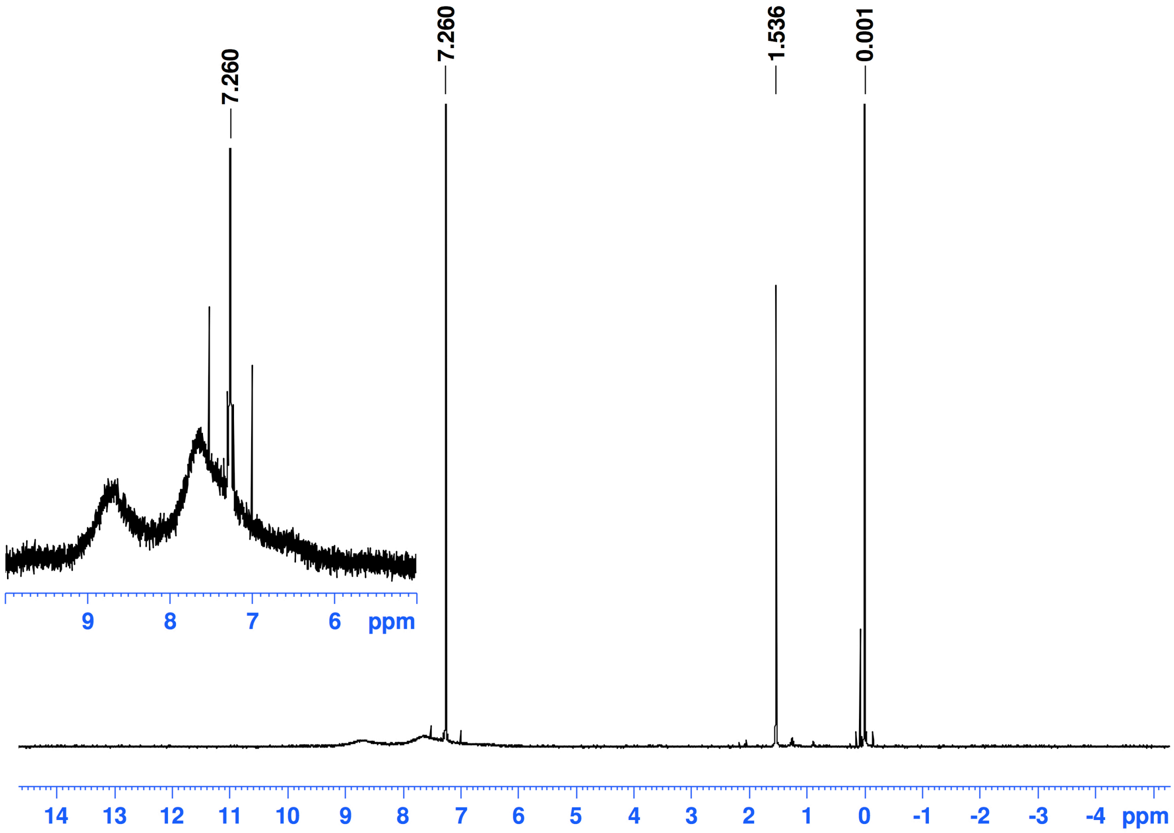

Supplementary Figure 15. ^{1}H NMR spectrum (400 MHz) of **22** in $CDCl_3$ at 25 °C.

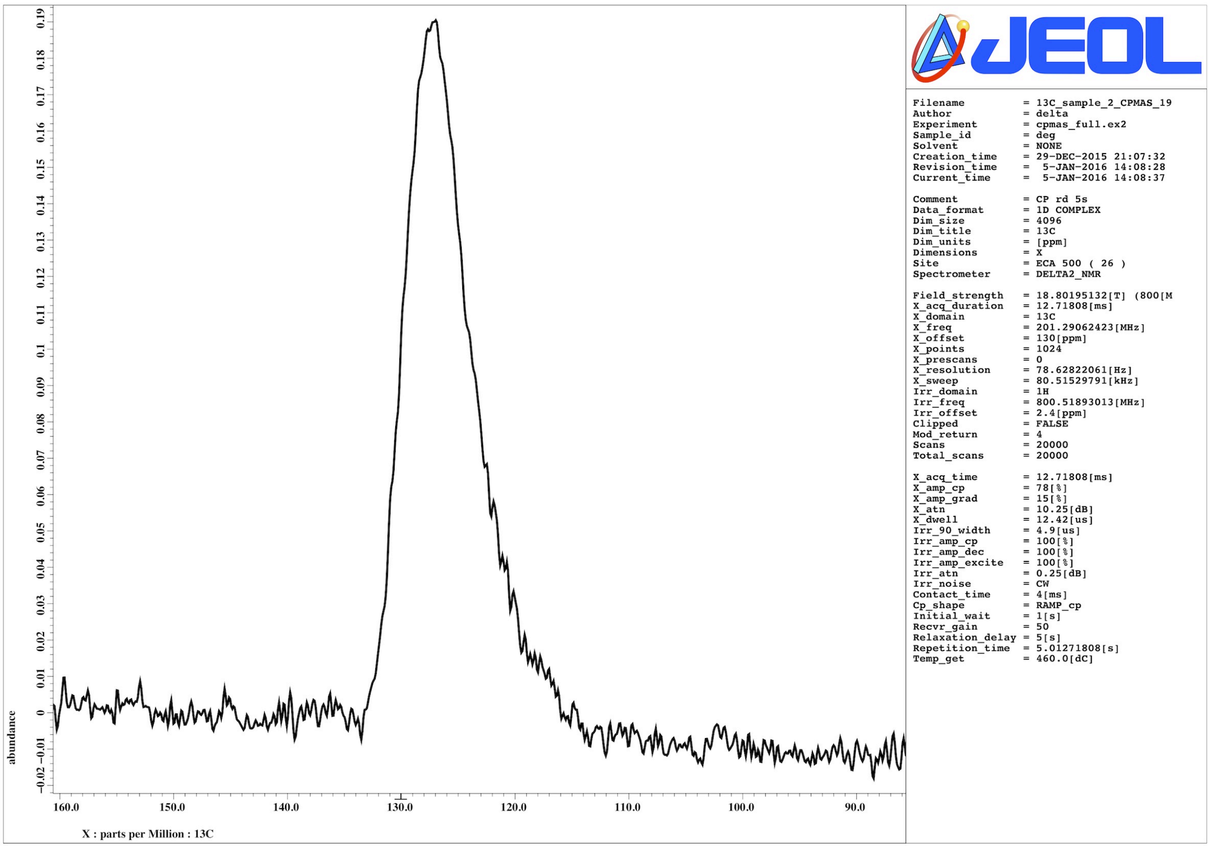

Supplementary Figure 16. CP/MAS ^{13}C NMR spectra of **22** (relaxation delay = 5 s) at 25 °C.

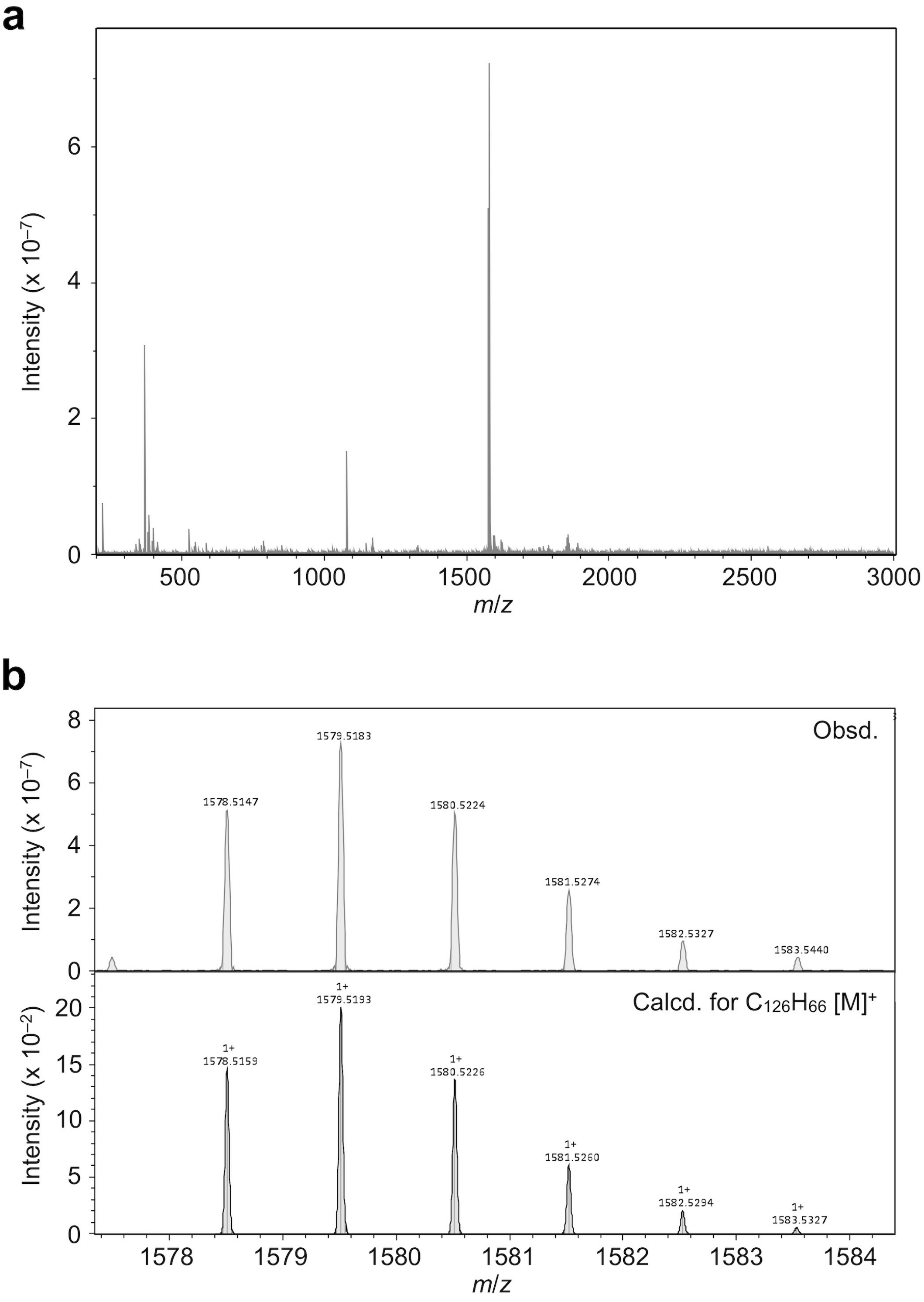

Supplementary Figure 17. FT-ICR MS spectrum of **22** (a) and magnifications for the area *m*/*z* = 1578–1584 (b). Sodium trifluoroacetate was used as the internal standard.

Supplementary Table 1. Crystal data for **3a**, **5** and **9**.

	**3a**	**5**	**9**
formula	$C_{26}H_{18}BCl$	$C_{28}H_{44}BClSi_4$	$C_{46}H_{26}$
FW	376.66	539.26	578.67
T/K	90	90	90
wavelength/Å	0.71073(Mo $K\alpha$)	0.71073(Mo $K\alpha$)	0.71073(Mo Kα)
color	pale yellow	colorless	orange-yellow
crystal size, mm	0.34 × 0.26 × 0.17	0.45 × 0.43 × 0.20	0.24 × 0.22 × 0.21
crystal system	orthorhombic	triclinic	monoclinic
space group	*Pbca* (#61)	*P*–1 (#2)	$P2_1/c$ (#14)
a/Å	8.7442(7)	9.3591(17)	10.2897(11)
b/Å	11.0103(9)	11.0339(19)	13.7936(10)
c/Å	39.240(3)	16.285(3)	20.341(2)
α/deg	90.000	88.004(3)	90.000
β/deg	90.000	80.264(3)	95.796(2)
γ/deg	90.000	70.252(2)	90.000
V/Å	3777.9(5)	1559.6(5)	2707.7(5)
Z	8	2	4
D_X/g cm^{-3}	1.324	1.148	1.420
μ/mm^{-1}	0.211	0.292	0.081
reflections collected	33854	8292	12735
unique reflections	3333	4942	4774
refined parameters	253	319	415
GOF on F^2	1.048	1.018	1.033
$R1$ [$I > 2\sigma(I)$][a]	0.0287	0.0503	0.0343
w$R2$ (all data)[b]	0.0675	0.1220	0.0898
$\Delta\rho_{min,\ max}$ / e Å^{-3}	−0.18, 0.26	−0.25, 0.30	−0.15, 0.28

[a] $R1$ = S ||Fo|–|Fc|| / S |Fo|, [b] w$R2$ = [S (w(Fo^2–Fc^2)^2/ S w(Fo^2)^2)$^{1/2}$

Supplementary Table 2. Crystal data for **12**, **14** and **20**.

	**12**	**14**	**20**
formula	$C_{46}H_{26}·C_7H_8$	$C_{66}H_{42}·0.5(C_6H_6)$	$C_{86}H_{46}·0.5(CS_2)$
FW	670.80	874.05	1117.37
T/K	90	90	93
wavelength/Å	0.71073(Mo *Kα*)	0.71073(Mo K*α*)	1.54187(Cu K*α*)
color	orange-yellow	colorless	orange
crystal size, mm	0.39 × 0.22 × 0.19	0.20 × 0.15 × 0.11	0.40 × 0.35 × 0.35
crystal system	monoclinic	triclinic	tetragonal
space group	$P2_1/c$ (#14)	*P*–1 (#2)	$P4_122$ (#91)
a/Å	14.8643(13)	10.7518(8)	34.605(7)
b/Å	13.4926(12)	13.7936(10)	34.605(7)
c/Å	16.3989(15)	17.8587(13)	35.904(8)
α/deg	90.000	74.1300(10)	90.000
β/deg	93.9330(10)	75.2430(10)	90.000
γ/deg	90.000	87.8050(10)	90.000
V/Å	3281.2(5)	2390.0(3)	42995(16)
Z	4	2	32
D_X/g cm^{-3}	1.358	1.215	1.378
μ/mm^{-1}	0.077	0.069	0.951
reflections collected	15483	23293	113441
unique reflections	5767	8403	37449
refined parameters	520	622	3138
GOF on F^2	1.052	1.037	1.281
$R1$ [$I > 2\sigma(I)$][a]	0.0607	0.0431	0.1543
w$R2$ (all data)[b]	0.1916	0.1175	0.4563
$\Delta\rho_{min,\ max}$ / e $Å^{-3}$	−0.74, 0.67	–0.38, 0.70	–0.67, 1.40

[a] $R1$ = S ||Fo|–|Fc|| / S |Fo|, [b] w$R2$ = [S (w(Fo^2–Fc^2)^2/ S w(Fo^2)^2)$^{1/2}$

Supplementary Table 3. One-electron oxidation of borepin **3a** using various oxidants.

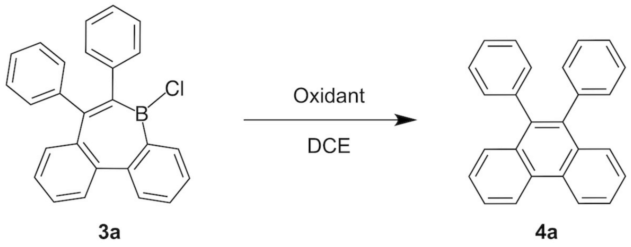

Entry	Oxidant	Reaction Time	Temperature	Yield of **4a**
1	O_2(g) (excess)	3 h	25 °C	54%* (59%)†
2	$[(p\text{-}BrC_6H_4)_3N^{\bullet+}][SbCl_6^-]$ (1.0 equiv.)	1 h	25 °C	64%* (86%)†
3	I_2 (1.5 equiv.)	24 h	60 °C	31%* (45%)†
4	$FeCl_3$ (1.0 equiv.)	1 h	25 °C	94%* (96%)†
5	MnO_2 (30 equiv.)	1 h	25 °C	94%* (99%)†
6	AZADO (1.0 equiv.)	1 h	25 °C	55%* (66%)†

*Isolated Yield. †Yield determined by ^{1}H NMR spectroscopy of the reaction mixture using hexamethylbenzene as the internal standard.

Supplementary Methods

General. Unless otherwise noted, all commercial reagents were used as received. Compounds **1**[2], **2b**–**2d**[3], **2e**[4], **2f**–**2h**[3], **10**[5], **13**[6], **15**[7], 2,2''-dibromo-1,1':2',1''-terphenyl[8] and 1-iodo-3-(phenylethynyl)benzene[9] were prepared according to previously reported procedures. Column chromatography was carried out using Wakogel silica C-200 (particle size: 75–150 μm). Preparative size-exclusion chromatography (SEC) was carried out on a Japan Analytical Industry LC-9210 NEXT recycling preparative HPLC system, equipped with JAIGEL-1HH and JAIGEL-2HH columns (diameter: 20 mm; length: 600 mm) and a multiwavelength detector (MD-2010$_{Plus}$) using $CHCl_3$ as the eluent. Melting points (m.p.) and decomposition points (d.p.) were recorded on a Yanaco MP-500D melting-point apparatus. Infrared (IR) spectra were recorded at 25 °C on a JASCO FT/IR-660$_{Plus}$ Fourier transform IR spectrometer. Nuclear magnetic resonance (NMR) spectroscopy measurements were carried out on a Bruker AVANCE-400 spectrometer (^{1}H: 400.0 MHz, ^{11}B: 128.3 MHz and ^{13}C: 100.6 MHz) or on a Bruker AVANCE III HD-500 spectrometer (^{1}H: 500.0 MHz, ^{13}C: 125.7 MHz, ^{11}B: 160.0 MHz and ^{29}Si: 99.0 MHz). Chemical shifts (δ) are expressed relative to the resonances of the residual non-deuterated solvent for ^{1}H ($CDCl_3$: ^{1}H(δ) = 7.26 ppm, ODCB-d_4: ^{1}H(δ) = 7.19 and 6.94 ppm), external $BF_3{\cdot}OEt_2$ in $CDCl_3$ for ^{11}B (^{11}B(δ) = 0.0 ppm), the resonances of the residual solvent for ^{13}C ($CDCl_3$: ^{13}C(δ) = 78.0 ppm, ODCB-d_4: ^{13}C(δ) = 132.2, 129.9, and 127.1 ppm) and internal tetramethylsilane in $CDCl_3$ for ^{29}Si (^{29}Si(δ) = 0.0 ppm). Absolute values of the coupling constants are given in Hertz (Hz), regardless of their sign. Multiplicities are abbreviated as singlet (s), doublet (d), triplet (t), quartet (q), multiplet (m) and broad (br). A JEOL ECA 800 spectrometer (201 MHz) equipped with a 3.2 mm MAS probehead was used for ^{13}C cross-polarization (CP) magic-angle spinning (MAS) NMR spectra at 25 °C, and chemical shifts (δ in ppm) are expressed with respect to adamantane as the external standard. A standard ramped ^{1}H–^{13}C CP sequence was used with a mixing time of 4 ms and MAS frequencies of 19–20 kHz. Mass spectrometry measurements were carried out on a Bruker micrOTOF II mass spectrometer equipped with an atmospheric pressure chemical ionization (APCI) probe or on a Bruker solariX XR 7.0T Fourier transform ion cyclotron resonance (FT-ICR) mass spectrometer.

Synthesis.

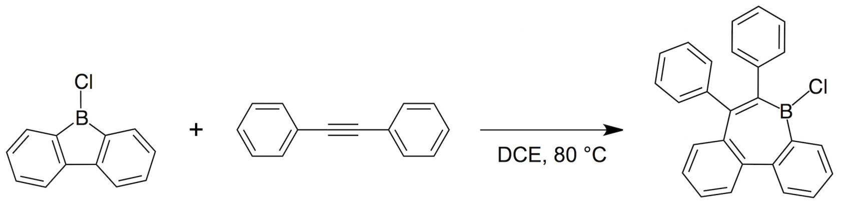

1 **2a** **3a**

Synthesis of 5-chloro-6,7-diphenyl-5*H*-dibenzo[*b,d*]borepin (3a). A dry DCE solution (2.0 mL) of a mixture of 9-chloro-9-borafluorene[2] **1** (109 mg, 0.55 mmol) and diphenylacetylene **2a** (89 mg, 0.50 mmol) was stirred for 12 h at 80 °C under argon and then evaporated to dryness. The residue was recrystallized from dry hexane to give **3a** as pale-yellow crystals (164 mg, 0.44 mmol) in 87% yield: m.p. (in a sealed tube under argon): 154 °C. FT-IR (ATR): ν (cm^{-1}) 2953, 2923, 2850, 1588, 1463, 1377, 1366, 1296, 1253, 1232, 1209, 1168, 1082, 1047, 1029, 951, 936, 914, 889, 786, 776, 759, 752, 740, 736, 721, 698, 664, 625. ^{1}H NMR (400 MHz, $CDCl_3$): δ (ppm) 7.93 (d, J = 7.9 Hz, 1H), 7.84 (dd, J = 7.5, 1.1 Hz, 1H), 7.75–7.65 (m, 2H), 7.49 (td, J = 14.9, 1.1 Hz, 1H), 7.37 (m, 1H), 7.25–7.17 (m, 2H), 7.16–6.96 (m, 8H), 6.96–6.88 (m, 2H). ^{13}C NMR (100 MHz, $CDCl_3$): δ (ppm) 152.2, 147.9, 144.5, 143.8, 143.2, 140.1, 139,3, 138.3, 132.4, 132.1, 132.0, 131.6, 130.5, 130.3, 129.3, 127.5 (two peaks), 127.2, 126.7, 126.6, 125.9; one peak of the aromatic carbon atom at the *ipso* position relative to the boron atom was not observed. ^{11}B NMR (128 MHz, $CDCl_3$): δ (ppm) 61.6. APCI-TOF MS: calcd. for $C_{26}H_{18}BCl$ $[M]^+$: m/z = 376.12; found: 376.12.

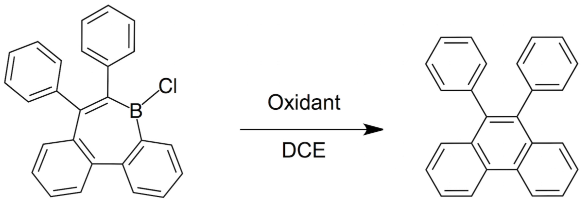

3a **4a**

One-electron oxidation of borepin 3a using various oxidants (see also Supplementary Table 3)

Oxidation with O_2. A dry DCE solution (2.0 mL) of **3a** (188 mg, 0.50 mmol) was degassed by three freeze–pump–thaw cycles and then exposed to 1 bar of O_2 (1.0 L) at 25 °C. After stirring for 3 h at 25 °C, the resulting mixture was passed through a plug of Florisil® and evaporated to dryness. The residue was subjected to SEC with $CHCl_3$ as the eluent, which allowed the isolation of 9,10-diphenylphenanthrene[10] **4a** as colorless crystals (89 mg, 0.27 mmol) in 54% yield: m.p. (in a sealed tube under argon): 242 °C. FT-IR (ATR): ν (cm^{-1}) 3100, 3056, 3048, 3027, 1606, 1584, 1575, 1527, 1487, 1441, 1419, 1321, 1139, 1073, 1047, 1028, 999, 885, 760, 750, 727, 701, 630. ^{1}H NMR (400 MHz, $CDCl_3$): δ (ppm) 8.81 (d, J = 8.2 Hz, 2H), 7.67 (ddd, J = 8.3, 7.7, 1.3 Hz, 2H), 7.56 (dd, J = 8.3, 1.2 Hz, 2H), 7.48 (ddd, J = 8.3, 7.7, 1.1 Hz, 2H), 7.12–7.28 (m, 10H). ^{13}C NMR (100 MHz, $CDCl_3$): δ (ppm) 139.7,

137.3, 132.0, 131.1, 130.1, 128.0, 127.7, 126.8, 126.6, 126.5, 122.6. APCI-TOF MS: calcd. for $C_{26}H_{18}$ $[M]^+$: m/z = 330.14; found: 330.14.

Oxidation with [(*p*-BrC$_6$H$_4$)$_3$N$^{•+}$][SbCl$_6^-$]. Under argon, $[(p\text{-}BrC_6H_4)_3N^{•+}][SbCl_6^-]$ (408 mg, 0.50 mmol) was added to a dry DCE solution (2.0 mL) of **3a** (188 mg, 0.50 mmol) at 25 °C. After stirring for 1 h at 25 °C, the resulting mixture was passed through a plug of Florisil® and evaporated to dryness. The residue was subjected to SEC with $CHCl_3$ as the eluent, which allowed the isolation of **4a** as colorless crystals (106 mg, 0.32 mmol) in 64% yield.

Oxidation with I$_2$. Under argon, I_2 (190 mg, 0.75 mmol) was added to a dry DCE solution (2.0 mL) of **3a** (188 mg, 0.50 mmol) at 25 °C. After stirring for 24 h at 60 °C, the resulting mixture was allowed to cool to 25 °C, poured into a saturated aqueous solution of sodium thiosulfate, and extracted with CH_2Cl_2. The organic layer was washed with water, dried over anhydrous Na_2SO_4, and then evaporated to dryness under reduced pressure. The obtained residue was dissolved in CH_2Cl_2, passed through a plug of Florisil®, and evaporated to dryness. The residue was subjected to SEC with $CHCl_3$ as the eluent, which allowed the isolation of **4a** as colorless crystals (51 mg, 0.16 mmol) in 31% yield.

Oxidation with FeCl$_3$. Under argon, a dry $MeNO_2$ solution (2.0 mL) of $FeCl_3$ (81 mg, 0.50 mmol) was added to a dry DCE solution (2.0 mL) of **3a** (188 mg, 0.50 mmol) at 25 °C. After stirring for 1 h at 25 °C, the resulting mixture was poured into MeOH (150 mL), diluted with water, and extracted with CH_2Cl_2. The organic layer was washed with water, dried over anhydrous Na_2SO_4, and evaporated to dryness under reduced pressure. The obtained residue was dissolved in CH_2Cl_2, passed through a plug of Florisil®, and evaporated to dryness, affording **4a** as colorless crystals (155 mg, 0.47 mmol) in 94% yield.

Oxidation with MnO$_2$. Under argon, MnO_2 (1.30 mg, 15.0 mmol) was added to a dry DCE solution (2.0 mL) of **3a** (188 mg, 0.50 mmol) at 25 °C. After stirring for 1 h at 25 °C, the resulting mixture was passed through a plug of Florisil® with CH_2Cl_2 as the eluent and then evaporated to dryness under reduced pressure to afford **4a** as colorless crystals (155 mg, 0.47 mmol) in 94% yield.

Oxidation with 2-azaadamantane-*N*-oxyl (AZADO). Under argon, AZADO (76 mg, 0.50 mmol) was added to a dry DCE solution (2.0 mL) of **3a** (188 mg, 0.50 mmol) at 25 °C. After stirring for 1 h at 25 °C, the resulting mixture was passed through a plug of Florisil® with CH_2Cl_2 as the eluent and then evaporated to dryness under reduced pressure. The obtained residue was subjected to SEC with $CHCl_3$ as the eluent, which allowed the isolation of **4a** as colorless crystals (91 mg, 0.28 mmol) in 55% yield.

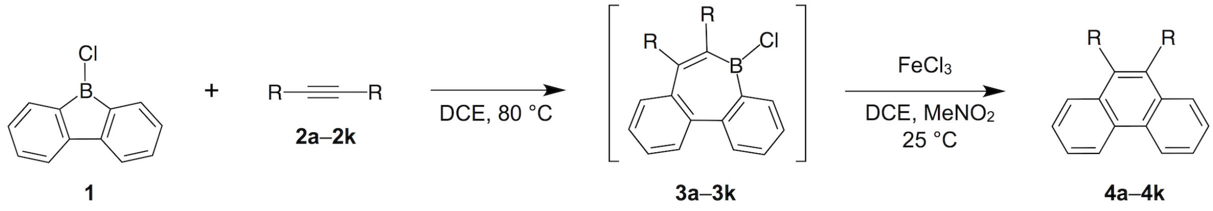

Synthesis of 9,10-bis(4-methylphenyl)phenanthrene (4b). According to the general procedure, **4b** was obtained as colorless crystals (153 mg, 0.44 mmol) in 85% yield from **1** and bis(4-mehtylphenyl)acetylene[3] (**2b**): m.p.: 245 °C. FT-IR (KBr): ν (cm^{-1}) 3064, 3045, 3020, 2920, 2864, 1955, 1927, 1901, 1632, 1615, 1605, 1527, 1508, 1488, 1447, 1418, 1377, 1322, 1213, 1182, 1165, 1139, 1108, 1045, 1021, 998, 974, 956, 888, 855, 841, 819, 798, 774, 761, 727, 655, 629. ^{1}H NMR (400 MHz, $CDCl_3$): δ (ppm) 8.80 (d, J = 8.4 Hz, 2H), 7.65 (dd, J = 8.7, 1.4 Hz, 2H), 7.56 (dd, J = 8.4, 1.1 Hz, 2H), 7.47 (dd, J = 8.6, 1.1 Hz, 2H), 7.05 (m, 8H), 2.33 (s, 6H). ^{13}C NMR (100 MHz, $CDCl_3$): δ (ppm) 137.2, 136.6, 135.8, 132.2, 130.9, 130.0, 128.4, 127.9, 126.5, 126.3, 122.5, 21.3. APCI-TOF MS: calcd. for $C_{28}H_{22}$ $[M]^+$: m/z = 358.17; found: 358.17.

Synthesis of 9,10-bis(4-methoxyphenyl)phenanthrene (4c). According to the general procedure, **4c** was obtained as colorless crystals (160 mg, 0.41 mmol) in 82% yield from **1** and bis(4-methoxyphenyl)acetylene[3] (**2c**): m.p.: 265 °C. FT-IR (KBr): ν (cm^{-1}) 3442, 3067, 3033, 3011, 2952, 2932, 2904, 2835, 2532, 2053, 2021, 1955, 1927, 1889, 1837, 1652, 1611, 1574, 1526, 1508, 1488, 1462, 1448, 1418, 1371, 1321, 1290, 1247, 1179, 1140, 1107, 1033, 1021, 998, 972, 958, 935, 888, 855, 833, 811, 796, 778, 761, 744, 727, 645, 627. ^{1}H NMR (400 MHz, $CDCl_3$): δ (ppm) 8.80 (d, J = 8.3 Hz, 2H,), 7.65 (dd, J = 8.5, 1.4 Hz, 2H), 7.59 (dd, J = 8.3, 1.1 Hz, 2H), 7.48 (dd, J = 8.4, 1.3 Hz, 2H), 7.06 (d, J = 8.7 Hz, 4H), 6.80 (d, J = 8.7 Hz, 4H), 3.81 (s, 6H). ^{13}C NMR (100 MHz, $CDCl_3$): δ (ppm) 158.0, 137.2, 132.3, 132.1, 132.0, 130.0, 127.9, 126.5, 126.2, 122.1, 113.1, 55.1. APCI-TOF MS: calcd. for $C_{28}H_{22}O_2$ $[M]^+$: m/z = 390.16; found: 390.16.

Synthesis of 9,10-bis(4-bromophenyl)phenanthrene (4d). According to the general procedure, **4d** was obtained as colorless crystals (200 mg, 0.41 mmol) in 82% yield from **1** and bis(4-bromophenyl)acetylene[3] (**2d**): m.p.: 271 °C. FT-IR (KBr): ν (cm^{-1}) 3086, 3065, 3041, 3024, 2955, 2925, 2853, 1956, 1925, 1905, 1896, 1732, 1596, 1525, 1486, 1467, 1447, 1418, 1390, 1322, 1212, 1138, 1099, 1072, 1045, 1012, 972, 886, 854, 830, 796, 759, 726, 699, 624, 574, 524. ^{1}H NMR (400 MHz, $CDCl_3$): δ (ppm) 8.81 (d, J = 8.3 Hz, 2H), 7.69 (dd, J = 8.32 and 2.18 Hz, 2H), 7.47–7.53 (m, 4H), 7.42 (dd, J = 8.32, 2.44 Hz, 4H), 7.02 (dd, J = 8.38, 2.46 Hz, 4H). ^{13}C NMR (100 MHz, $CDCl_3$): δ (ppm) 138.2, 136.0, 132.6, 131.4, 131.1, 130.1, 127.6, 126.8 (two peaks), 122.6, 121.0. APCI-TOF MS: calcd. for $C_{26}H_{16}Br_2$ $[M]^+$: m/z = 485.96; found: 485.96.

Synthesis of 9,10-bis(4-iodophenyl)phenanthrene (4e). According to the general procedure, **4e** was obtained as colorless crystals (232 mg, 0.40 mmol) in 80% yield from **1** and bis(4-

iodophenyl)acetylene[4] (**2e**): m.p.: 270 °C. FT-IR (KBr): ν (cm^{-1}) 3068, 3039, 3024, 1954, 1899, 1726, 1609, 1592, 1525, 1483, 1447, 1418, 1387, 1319, 1238, 1210, 1181, 1166, 1138, 1099, 1061, 1047, 1007, 971, 884, 854, 823, 793, 759, 726, 693, 675, 637, 624. ^{1}H NMR (400 MHz, $CDCl_3$): δ (ppm) 8.80 (d, J = 8.4 Hz, 2H), 7.67–7.70 (m, 2H), 7.61 (d, J = 8.0 Hz, 4H), 7.47–7.52 (m, 4H), 6.89 (d, J = 8.0 Hz, 4H). ^{13}C NMR (100 MHz, $CDCl_3$): δ (ppm) 138.8, 137.0, 136.0, 132.9, 131.4, 130.1, 127.6, 126.9, 126.8, 122.6, 92.7. APCI-TOF MS: calcd. for $C_{26}H_{16}I_2$ $[M]^+$: m/z = 581.93; found: 581.93.

Synthesis of 9,10-bis(4-trifluoromethylphenyl)phenanthrene (4f). According to the general procedure, **4f** was obtained as colorless crystals (157 mg, 0.34 mmol) in 67% yield from **1** and bis(4-trifluoromethylphenyl)acetylene[3] (**2f**): m.p.: 292 °C. FT-IR (KBr): ν (cm^{-1}) 3072, 3048, 1928, 1918, 1619, 1582, 1491, 1449, 1420, 1408, 1327, 1239, 1212, 1163, 1125, 1107, 1067, 1046, 1021, 974, 953, 890, 866, 845, 813, 766, 751, 728, 688, 673, 637, 607. ^{1}H NMR (400 MHz, $CDCl_3$): δ (ppm) 8.84 (d, J = 8.4 Hz, 2H), 7.72 (dd, J = 8.4 Hz, 1.3, 2H), 7.51–7.55 (m, 6H), 7.44 (d, J = 8.5 Hz, 2H), 7.28 (d, J = 7.9 Hz, 4H). ^{13}C NMR (100 MHz, $CDCl_3$): δ (ppm) 143.0, 136.0, 131.3, 131.1, 130.2, 129.2 (q, J = 32.8 Hz), 127.5, 127.1, 125.2, 124.9 (q, J = 3.42 Hz), 123.0, 122.7, 120.9 (two peaks). APCI-TOF MS: calcd. for $C_{28}H_{16}F_6$ $[M]^+$: m/z = 466.11; found: 466.12.

Synthesis of 9,10-bis(4-methoxycarbonylphenyl)phenanthrene (4g). According to the general procedure, **4g** was obtained as colorless crystals (107 mg, 0.24 mmol) in 48% yield from **1** and bis(4-methyoxycarbonylphenyl)acetylene[3] (**2g**): d.p.: 323 °C. FT-IR (KBr): ν (cm^{-1}) 3068, 3032, 3003, 2952, 2900, 2841, 2584, 2557, 2065, 1974, 1936, 1730, 1715, 1608, 1586, 1563, 1528, 1507, 1489, 1447, 1435, 1420, 1404, 1370, 1289, 1214, 1192, 1179, 1141, 1115, 1104, 1045, 1020, 960, 875, 862, 825, 814, 777, 765, 728, 711, 698, 642, 626, 615. ^{1}H NMR (400 MHz, $CDCl_3$): δ (ppm) 8.82 (d, J = 8.4 Hz, 2H), 7.92 (d, J = 8.1 Hz, 4H), 7.70 (dd, J = 8.5, 1.4 Hz, 2H), 7.45–7.52 (m, 4H), 7.24 (d, J = 8.2 Hz, 4H), 3.91 (s, 6H). ^{13}C NMR (100 MHz, $CDCl_3$): δ (ppm) 167.0, 144.3, 136.2, 131.1, 130.1, 129.1, 128.7, 127.5, 126.9 (two peaks), 122.7, 52.1. APCI-TOF MS: calcd. for $C_{30}H_{22}O_4$ $[M]^+$: m/z = 446.15; found: 446.15.

Synthesis of 9,10-di(thiophene-2-yl)phenanthrene (4h). According to the general procedure, **4h** was obtained as colorless crystals (125 mg, 0.36 mmol) in 73% yield from **1** and di(thiophene-2-yl)acetylene[3] (**2h**): m.p.: 242 °C. FT-IR (KBr): ν (cm^{-1}) 3102, 3069, 1961, 1933, 1723,1607, 1582, 1535, 1489, 1449, 1435, 1350, 1304, 1228, 1212, 1175, 1163, 1126, 1117, 1090, 1035, 956, 862, 853, 831, 764, 726, 695, 655, 616. ^{1}H NMR (400 MHz, $CDCl_3$): δ (ppm) 8.78 (d, J = 8.3 Hz, 2H), 7.81 (dd, J = 8.4, 1.1 Hz, 2H), 7.70 (dd, J = 8.4, 1.4 Hz, 2H), 7.55 (dd, J = 8.2, 1.2 Hz, 2H), 7.34 (dd, J = 5.1, 1.2 Hz, 2H), 7.02 (dd, J = 5.1, 3.5 Hz, 2H), 6.95 (dd, J = 3.5, 1.2 Hz, 2H). ^{13}C NMR (100 MHz, $CDCl_3$): δ (ppm) 139.8, 132.3, 132.2, 130.3, 129.4, 127.9, 127.2, 126.9, 126.4, 126.2, 122.5. APCI-TOF MS: calcd. for $C_{22}H_{14}S_2$ $[M]^+$: m/z = 342.05; found: 342.05.

Synthesis of 9-ethyl-10-phenylphenanthrene (4i). According to the general procedure, **4i** was obtained as colorless crystals (90 mg, 0.32 mmol) in 64% yield from **1** and 1-phenyl-1-butyne: m.p.: 158 °C. FT-IR (KBr): ν (cm^{-1}) 3073, 3024, 2965, 2928, 2874, 1602, 1587, 1491, 1448, 1421, 1378, 1366, 1314, 1238, 1063, 1043, 1031, 1026, 1002, 954, 896, 840, 785, 762, 726, 705, 632, 617, 607. ^{1}H NMR (400 MHz, $CDCl_3$): δ (ppm) 8.77–8.85 (m, 1H), 8.74 (d, J = 8.4 Hz, 1H), 8.15–8.24 (m, 1H), 7.65–7.72 (m, 2H), 7.45–7.62 (m, 4H), 7.39–7.45 (m, 1H), 7.29–7.37 (m, 3H), 2.91 (q, J = 7.5 Hz, 2H), 1.22 (t, J = 7.5 Hz, 3H). ^{13}C NMR (100 MHz, $CDCl_3$): δ (ppm) 140.7, 136.8, 136.1, 132.6, 130.8, 130.6, 130.3, 129.5, 128.5, 127.7, 127.2, 126.9, 126.4, 126.2, 125.8, 125.3, 123.3, 122.4, 23.7, 15.4. APCI-TOF MS: calcd. for $C_{22}H_{18}$ $[M]^+$: m/z = 282.14; found: 282.14.

Synthesis of 9,10-diethylphenanthrene (4j). According to the general procedure, **4j** was obtained as colorless crystals (83 mg, 0.36 mmol) in 71% yield from **1** and 3-hexyne: m.p.: 106 °C. FT-IR (KBr): ν (cm^{-1}) 3074, 2970, 2929, 2872, 1607, 1585, 1495, 1449, 1422, 1376, 1304, 1239, 1052, 908, 757, 726, 617. ^{1}H NMR (400 MHz, $CDCl_3$): δ (ppm) 8.68–8.78 (m, 2H), 8.10–8.17 (m, 2H), 7.55–7.67 (m, 4H), 3.22 (q, J = 7.6 Hz, 4H), 1.38 (t, J = 7.6 Hz, 6H). ^{13}C NMR (100 MHz, $CDCl_3$): δ (ppm) 135.0, 131.3, 130.0, 126.7, 125.5, 124.7, 123.1, 22.3, 15.2. APCI-TOF MS: calcd. for $C_{18}H_{18}$ $[M]^+$: m/z = 234.14; found: 234.13.

Synthesis of 9-phenylphenanthrene (4k). According to the general procedure, **4k** was obtained as colorless crystals (55 mg, 0.22 mmol) in 34% yield from **1** and phenylacetylene: m.p.: 110 °C. FT-IR (KBr): ν (cm^{-1}) 3059, 1594, 1492, 1450, 1378, 1248, 1230, 1151, 1138, 1070, 1029, 892, 867, 777, 769, 749, 725, 700, 616. ^{1}H NMR (400 MHz, $CDCl_3$): δ (ppm) 8.80 (d, J = 8.6 Hz, 1H), 8.75 (d, J = 8.3 Hz, 1H), 7.88–7.98 (m, 2H), 7.60–7.74 (m, 4H), 7.45–7.60 (m, 6H). ^{13}C NMR (100 MHz, $CDCl_3$): δ (ppm) 140.8, 138.8, 131.6, 131.2, 130.7, 130.1, 130.0, 128.7, 128.3, 127.5, 127.4, 127.0, 126.9, 126.6, 126.5, 126.5, 122.9, 122.6. APCI-TOF MS: calcd. for $C_{20}H_{14}$ $[M]^+$: m/z = 254.11; found: 254.11.

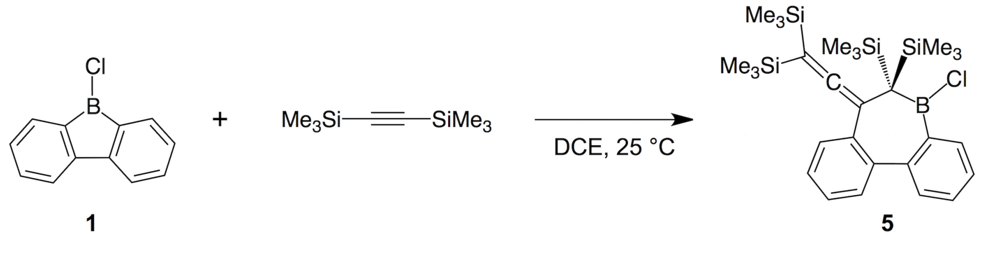

Synthesis of 5. Under argon, a dry DCE solution (1.0 mL) of a mixture of **1** (110 mg, 0.55 mmol) and bis(trimethylsilyl)acetylene (206 mg, 1.2 mmol) was stirred for 12 h at 25 °C. The resulting mixture was evaporated to dryness under reduced pressure. The obtained residue was recrystallized from dry hexane to give **5** as colorless crystals (264 mg, 0.49 mmol) in 89% yield: d.p.: 158 °C. FT-IR (ATR): ν (cm^{-1}) 3055, 2956, 2926, 2894, 1883, 1590, 1434, 1411, 1291, 1242, 1217, 1190, 1089, 1075, 1041, 973, 905, 878, 839, 793, 782, 760, 749, 739, 716, 686, 647, 633, 610. ^{1}H NMR (500 MHz, $CDCl_3$): δ (ppm) 7.66 (dd, J = 7.4, 1.3 Hz, 1H),

7.48–7.42 (m, 2H), 7.38–7.29 (m, 4H), 7.27–7.23 (m, 1H), 0.32 (s, 9H), 0.24 (s, 9H), 0.02 (s, 9H), –0.35 (s, 9H). ^{13}C NMR (125 MHz, $CDCl_3$): δ (ppm) 209.3, 144.4, 142.7, 141.0, 132.4, 131.2, 130.9, 129.9, 129.7, 127.7, 127.3, 126.4, 91.7, 89.8, 3.6, 3.4, 1.3, 0.28; the peak for the aromatic carbon atom at the *ipso* position relative to the boron atom, was not observed. ^{11}B NMR (160 MHz, $CDCl_3$): δ (ppm) 68.3. ^{29}Si NMR (99 MHz, $CDCl_3$): δ (ppm) 3.6, –0.3. APCI-TOF MS: calcd. for $C_{28}H_{44}BClSi_4$ $[M]^+$: *m/z* = 538.24; found: 538.24.

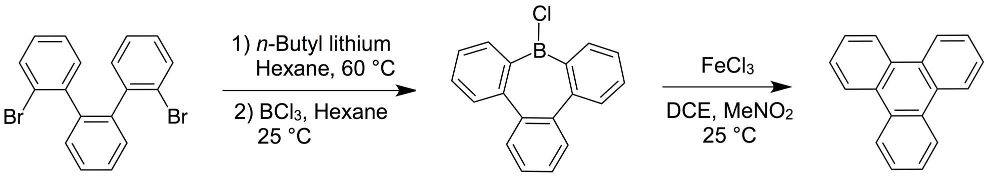

6

Synthesis of 9-chloro-9*H*-tribenzo[*b*,*d*,*f*]borepin (6). Under argon, a dry hexane solution of *n*-butyl lithium (2.6 M, 0.65 mL) was added dropwise to a dry hexane solution (5.0 mL) of 2,2”-dibromo-1,1’:2’,1”-terphenyl[8] (300 mg, 0.77 mmol) at 0 °C. This mixture was stirred for 48 h at 60 °C and then allowed to cool to 25 °C. After the addition of a hexane solution of boron trichloride (1.0 M, 0.80 mL), the reaction mixture was stirred for 24 h at 25 °C and then evaporated to dryness under reduced pressure. The obtained residue was dissolved in hexane, passed through a plug of celite®, and evaporated to dryness. The resulting residue was recrystallized from hexane at –35 °C to give **6** as a colorless solid (68 mg, 0.25 mmol) in 32% yield: d.p.: 126 °C. FT-IR (ATR): ν (cm^{-1}) 3054, 1586, 1553, 1468, 1428, 1303, 1293, 1256, 1173, 930, 908, 781, 764, 741, 722, 664, 617, 604. ^{1}H NMR (400 MHz, $CDCl_3$): δ (ppm) 8.03 (dd, J = 7.6, 1.2 Hz, 2H), 7.74 (d, J = 7.9 Hz, 2H), 7.63 (dd, J = 3.52, 2.30 Hz, 2H), 7.60 (d, J = 7.7 Hz, 2H), 7.48 (dd, J = 3.52, 2.30 Hz, 2H), 7.45 (d, J = 7.7 Hz, 2H). ^{13}C NMR (100 MHz, $CDCl_3$): δ (ppm) 144.4, 143.2 (br), 138.6, 132.4, 132.2, 132.0, 129.4, 127.7, 126.9. ^{11}B NMR (128 MHz, $CDCl_3$): δ (ppm) 62.5. APCI-TOF MS: calcd. for $C_{18}H_{12}BCl$ $[M]^+$: *m/z* = 274.07; found: 274.08.

Oxidation of 6 with $FeCl_3$. A dry $MeNO_2$ solution (1.0 mL) of $FeCl_3$ (30 mg, 0.50 mmol) was added to a dry DCE solution (1.0 mL) of **6** (50 mg, 0.18 mmol) at 25 °C. After stirring for 30 min at 25 °C, the resulting mixture was poured into MeOH (75 mL) and partially evaporated under reduced pressure until the volume of the solution was reduced to ~30 mL. The resulting solution was poured into water and extracted with CH_2Cl_2. The organic layer was washed with water, dried over anhydrous Na_2SO_4, and evaporated to dryness under reduced pressure. The obtained residue was dissolved in CH_2Cl_2, passed through a plug of Florisil®, and evaporated to dryness under reduced pressure, which afforded triphenylene as colorless crystals (41 mg, 0.18 mmol) in 99% yield.

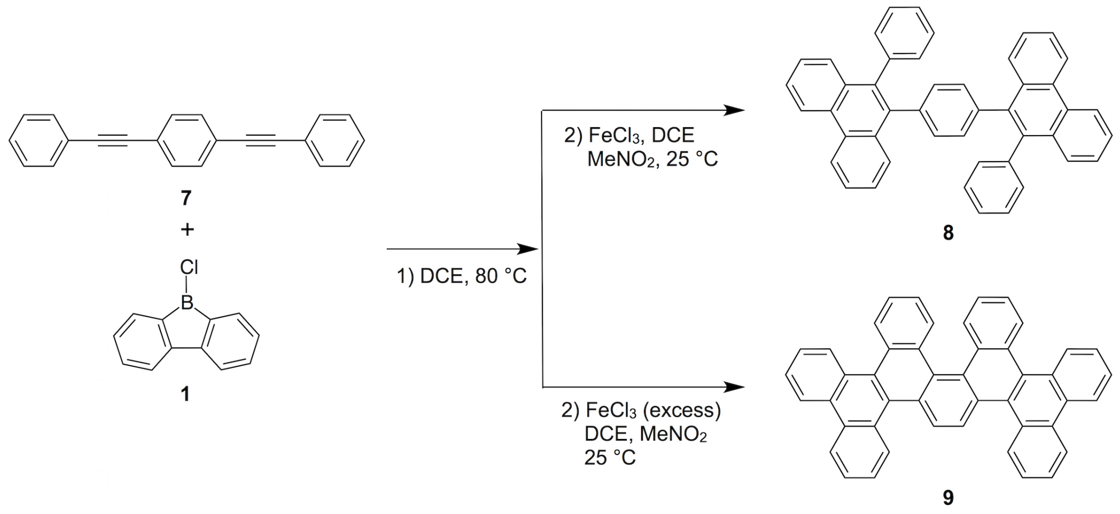

Synthesis of 1,4-bis(10-phenylphenanthren-9-yl)benzene (8). Under argon, a dry DCE solution (1.5 mL) of a mixture of **1** (157 mg, 0.79 mmol) and 1,4-bis(phenylethynyl)benzene (**7**; 100 mg, 0.36 mmol) was stirred for 24 h at 80 °C and then allowed to cool to 25 °C. After the addition of a dry $MeNO_2$ solution (1.0 mL) of $FeCl_3$ (116 mg, 0.72 mmol), the reaction mixture was stirred for 1 h at 25 °C, and subsequently poured into MeOH (80 mL). The precipitate thus formed was collected by filtration, dissolved in CH_2Cl_2, passed through a plug of Florisil®, and evaporated to dryness under reduced pressure, affording **8** as colorless crystals (184 mg, 0.32 mmol) in 88% yield: d.p.: 371 °C. FT-IR (KBr): ν (cm^{-1}) 3062, 3024, 2880, 1609, 1588, 1528, 1511, 1487, 1446, 1418, 1401, 1352, 1321, 1308, 1286, 1237, 1167, 1141, 1103, 1000, 972, 948, 916, 884, 858, 807, 794, 760, 741, 726, 700, 672, 639, 605. ^{1}H NMR (500 MHz, $CDCl_3$): δ (ppm) 8.83–8.80 (m, 4H), 7.70–7.57 (m, 7H), 7.53–7.46 (m, 4H), 7.35–7.29 (m, 4H), 7.24–7.20 (m, 5H), 7.09–7.07 (m, 3H), 7.02–7.00 (m, 3H). ^{13}C NMR (125 MHz, $CDCl_3$): δ (ppm) 139.7, 137.7, 137.2, 131.3, 130.8, 130.4, 130.3, 130.0 (two peaks), 127.9, 127.8, 126.8, 127.6, 126.7, 126.6 (two peaks), 126.3 (two peaks), 122.5, 122.4. APCI-TOF MS: calcd. for $C_{46}H_{30}$ $[M]^+$: m/z = 582.23; found: 582.23.

Synthesis of hexabenzo[*a*,*c*,*f*,*j*,*m*,*o*]picene (9). A dry DCE solution (1.5 mL) of **1** (157 mg, 0.79 mmol) and 1,4-bis(phenylethynyl)benzene (**7**; 100 mg, 0.36 mmol) was stirred for 24 h at 80 °C and then allowed to cool to 25 °C. CH_2Cl_2 (100 mL) and a dry $MeNO_2$ solution (1.0 mL) of $FeCl_3$ (1.75 g, 10.8 mmol) were added successively to the reaction mixture under argon bubbling through a glass capillary. After stirring for 1 h at 25 °C, the resulting mixture was poured into MeOH (150 mL). The yellow precipitate thus formed was collected by filtration, dissolved in $CHCl_3$, passed through a plug of Florisil®, and then evaporated to dryness under reduced pressure. The residue was recrystallized from a mixture of $CHCl_3$ and MeOH to afford **9** as orange crystals (185 mg, 0.32 mmol) in 89% yield: d.p.: 437 °C. FT-IR (KBr): ν (cm^{-1}) 3069, 3025, 1605, 1574, 1561, 1533, 1491, 1462, 1444, 1430, 1419, 1376, 1347, 1330, 1214, 1168, 1119, 1102, 1069, 1049, 1033, 977, 949, 925, 892, 798, 786, 757, 727, 714, 687, 676, 641, 630, 618, 610. ^{1}H NMR (500 MHz, $CDCl_3$): δ (ppm) 9.02 (s, 2H), 8.96–8.94 (m, 2H), 8.84 (dd, J = 8.0, 1.3 Hz, 2H), 8.78–8.76 (m, 4H), 8.67 (d, J = 8.0 Hz, 2H),

8.40 (d, J = 8.0 Hz, 2H), 7.78–7.68 (m, 8H), 7.53 (t, J = 8.0 Hz, 2H), 7.30 (t, J = 8.0 Hz, 2H). ^{13}C NMR (125 MHz, $CDCl_3$): δ (ppm) 131.3 (two peaks), 131.2, 130.1, 129.5, 129.2, 129.0 (two peaks), 128.9 (two peaks), 128.5 (two peaks), 127.3, 126.9, 126.8, 126.6, 126.5, 126.4, 126.2, 126.1, 124.7, 123.7, 123.5. APCI-TOF MS: calcd. for $C_{46}H_{26}$ $[M]^+$: m/z = 578.20; found: 578.20.

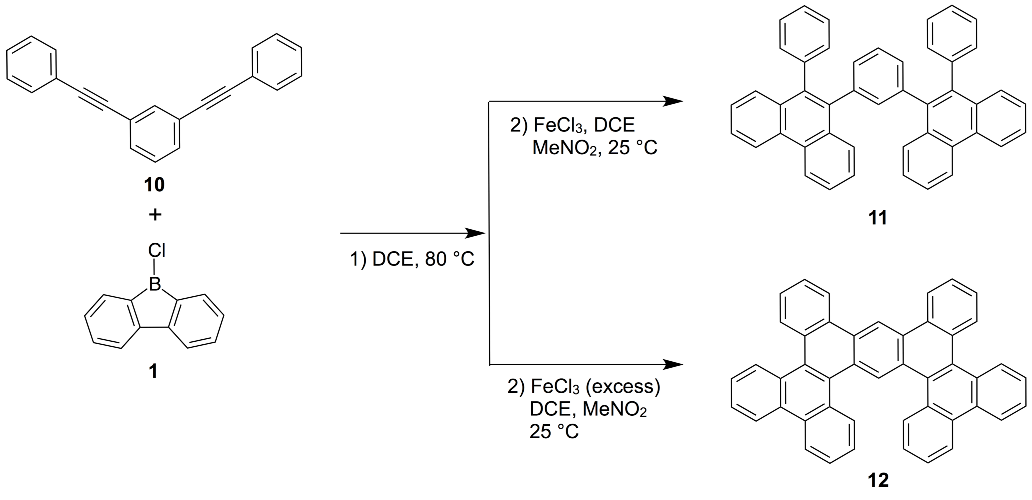

Synthesis of 1,3-bis(10-phenylphenanthren-9-yl)benzene (11). Using a procedure similar to that for **8**, **11** was obtained as colorless crystals in 89% yield from **1** and 1,3-bis(phenylethynyl)benzene[5] (**10**): m.p.: 281 °C. FT-IR (KBr): ν (cm^{-1}) 3063, 3023, 2977, 2940, 2896, 1946, 1928, 1891, 1815, 1603, 1581, 1526, 1482, 1445, 1418, 1374, 1320, 1307, 1283, 1262, 1237, 1161, 1115, 1085, 1070, 1046, 1030, 1010, 949, 928, 912, 857, 799, 760, 726, 701, 674, 659, 623. ^{1}H NMR (500 MHz, $CDCl_3$): δ (ppm) 8.78 (d, J = 8.5 Hz, 4H), 7.70–7.63 (m, 4H), 7.56 (dd, J = 8.4, 1.2 Hz, 2H), 7.50–7.45 (m, 4H), 7.35–7.33 (m, 4H), 7.30–7.22 (m, 8H), 7.14–7.08 (m, 4H), 6.99-6.96 (m, 4H). ^{13}C NMR (125 MHz, $CDCl_3$): δ (ppm) 139.6, 139.2, 137.2, 137.1, 133.2, 131.9, 131.7, 131.3, 131.0, 129.9, 129.8, 129.0, 128.0, 127.7 (two peaks), 127.1, 126.7, 126.6, 126.5, 126.3, 122.5, 122.3. APCI-TOF MS: calcd. for $C_{46}H_{30}$ $[M]^+$: m/z = 582.23; found: 582.23.

Synthesis of tetrabenzo[*a,c,f,k*]phenanthro[9,10-*m*]tetraphene (12). Using a procedure similar to that for **9**, **12** was obtained as orange crystals in 85% yield from **1** and 1,3-bis(phenylethynyl)benzene[5] (**10**): m.p.: >440 °C. FT-IR (KBr): ν (cm^{-1}) 3066, 3020, 1607, 1561, 1552, 1529, 1510, 1482, 1428, 1374, 1321, 1215, 1165, 1052, 1003, 945, 927, 883, 793, 759, 729, 713, 703, 677, 661. ^{1}H NMR (400 MHz, $CDCl_3$): δ (ppm) 9.89 (s, 1H), 9.65 (s, 1H), 9.02 (d, J = 8.0 Hz, 2H), 8.90 (d, J = 8.0 Hz, 2H), 8.63–8.74 (m, 8H), 7.74–7.84 (m, 4H), 7.67–7.74 (m, 4H), 7.65 (dd, J = 14.9, 7.8 Hz, 2H), 7.60 (dd, J = 14.9, 7.8 Hz, 2H). ^{13}C NMR (100 MHz, ODCB-d_4): δ (ppm) 131.5, 130.9, 130.8, 129.1 (two peaks), 129.0 (two peaks), 128.7, 128.6, 128.3, 128.0, 127.6, 126.7, 126.6 (two peaks), 124.4, 123.6, 123.5 (other peaks overlapped with those of the residual solvent). APCI-TOF mass: calcd. for $C_{46}H_{26}$ $[M]^+$: m/z = 578.23; found: 578.23.

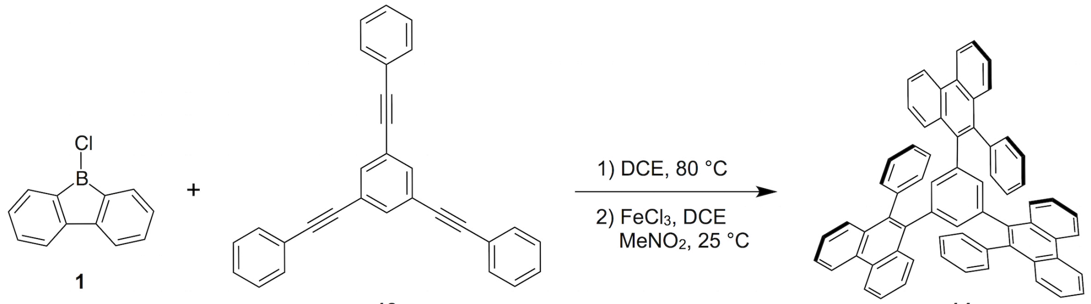

1 13 14

Synthesis of 1,3,5-tris(10-phenylphenanthren-9-yl)benzene (14). Under argon, a dry DCE solution (1.5 mL) of a mixture of **1** (218 mg, 1.1 mmol) and 1,3,5-tris(phenylethynyl)benzene[6] (**13**; 126 mg, 0.33 mmol) was stirred for 24 h at 80 °C and then allowed to cool to 25 °C. After the addition of a dry $MeNO_2$ solution (1.0 mL) of $FeCl_3$ (162 mg, 1.0 mmol), the reaction mixture was stirred for 1 h at 25 °C and then poured into MeOH (150 mL). The yellow precipitate thus formed was collected by filtration and subjected to column chromatography on SiO_2 (CH_2Cl_2/hexane; v/v = 1/2), which allowed the isolation of **14** as a pale-yellow powder (102 mg, 0.12 mmol) in 35% yield: m.p.: 240 °C. FT-IR (KBr): ν (cm^{-1}) 3070, 3025, 2592, 2924, 2868, 2851, 1598, 1578, 1560, 1542, 1524, 1488, 1447, 1418, 1363, 1313, 1138, 1072, 1048, 1035, 1001, 949, 934, 908, 892, 841, 755, 702, 619. ^{1}H NMR (500 MHz, $CDCl_3$): δ (ppm) 8.76–8.73 (m, 6H), 7.70–7.66 (m, 3H), 7.63–7.59 (m, 3H), 7.56–7.52 (m, 3H), 7.48–7.30 (m, 13H), 7.22–7.20 (m, 2H), 7.16–7.10 (m, 6H), 6.93 (t, J = 1.6 Hz, 1H), 6.90–6.88 (m, 2H), 6.78 (d, J = 1.6 Hz, 2H), 6.47 (dd, J = 8.0, 1.1 Hz, 1H). ^{13}C NMR (125 MHz, $CDCl_3$): δ (ppm) 139.8, 139.3, 139.2, 138.5, 137.3, 137.1, 136.7, 136.3, 132.1, 131.8 (two peaks), 131.7 (two peaks), 131.6, 131.4, 131.3, 130.6, 130.0, 129.9 (two peaks), 128.6, 128.2, 128.0, 127.9, 127.7 (two peaks), 127.6, 126.7, 126.6 (two peaks), 126.5 (two peaks), 126.3 (two peaks), 126.2 (two peaks), 122.4 (two peaks), 122.3, 121.9. APCI-TOF MS: calcd. for $C_{66}H_{42}$ $[M]^+$: m/z = 834.33; found: 834.33.

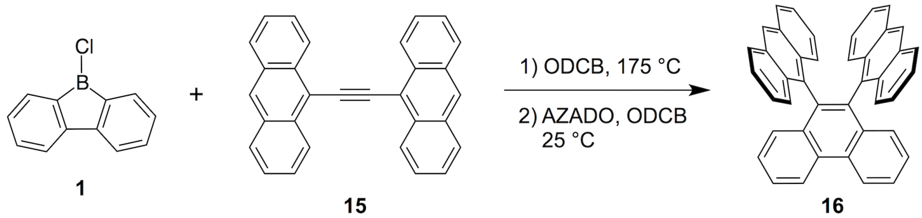

1 15 16

Synthesis of 9,10-di(anthracen-9-yl)phenanthrenes (16). Under argon, a dry ODCB suspension (8.0 mL) of **1** (892 mg, 4.5 mmol) and di(anthracen-9-yl)acetylene[7] (**15**; 300 mg, 0.79 mmol) was stirred for 96 h at 175 °C and then allowed to cool to 25 °C. After the addition of AZADO (137 mg, 0.90 mmol), the reaction mixture was stirred for 30 min at 25 °C, poured into saturated aqueous $NaHCO_3$, and then extracted with CH_2Cl_2. The organic layer was washed successively with water and brine, dried over anhydrous $MgSO_4$, and

evaporated to dryness under reduced pressure. The obtained residue was washed with hexane and recrystallized from DCE to give **16** as yellow crystals (172 mg, 0.32 mmol) in 40% yield: m.p.: 339 °C (sublim.). FT-IR (KBr): ν (cm^{-1}) 3069, 3434, 3047, 1623, 1519, 1487, 1443, 1415, 1298, 1160, 1013, 956, 889, 863, 788, 759, 730, 670, 617, 589. ^{1}H NMR (500 MHz, CD_2Cl_2): δ (ppm) 9.08 (d, J = 8.4 Hz, 2H), 8.00 (s, 2H), 7.79 (t, J = 7.7 Hz, 2H), 7.64 (d, J = 8.6 Hz, 4H), 7.58 (d, J = 8.8 Hz, 4H), 7.36 (t, J = 7.6 Hz, 2H), 7.17 (d, J = 8.2 Hz, 2H), 7.13 (J = 7.7 Hz, 4H), 6.90 (t, J = 7.8 Hz, 4H). ^{13}C NMR (125 MHz, $CDCl_3$): δ (ppm) 137.0, 133.6, 133.5, 131.1, 130.8, 130.4, 128.4, 128.2, 128.0, 127.6, 127.5, 126.6, 125.0, 124.9, 123.3. APCI-TOF MS: calcd. for $C_{42}H_{26}$ $[M]^+$: m/z = 530.20; found: 530.20.

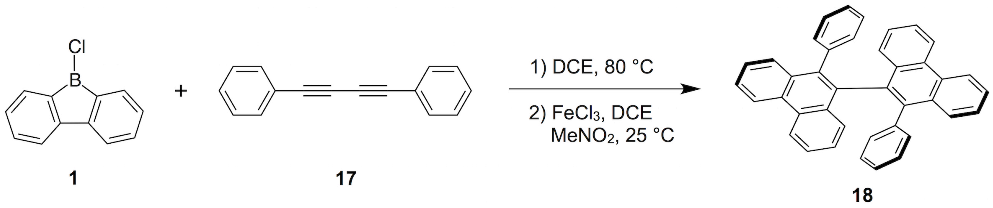

Synthesis of 10,10'-diphenyl-9,9'-biphenanthrene (18). Under argon, a dry DCE solution (1.0 mL) of a mixture of **1** (109 mg, 0.55 mmol) and 1,4-diphenylbutadiyne (**17**; 51 mg, 0.25 mmol) was stirred for 3 days at 80 °C and then allowed to cool to 25 °C. After the addition of a dry $MeNO_2$ solution (1.0 mL) of $FeCl_3$ (82 mg, 0.51 mmol), the reaction mixture was stirred for 1 h at 25 °C, poured into MeOH (150 mL), diluted with water, and extracted with CH_2Cl_2. The organic layer was washed with water, dried over anhydrous Na_2SO_4, and evaporated to dryness under reduced pressure. The obtained residue was subjected to column chromatography on SiO_2 (CH_2Cl_2/hexane; v/v = 1/2), which allowed the isolation of **18** as a white powder (100 mg, 0.20 mmol) in 80% yield: m.p.: 308 °C. FT-IR (KBr): ν (cm^{-1}) 3067, 3029, 2960, 2927, 2853, 1950, 1807, 1727, 1605, 1576, 1523, 1487, 1445, 1418, 1261, 1097, 1074, 1044, 1023, 913, 866, 803, 761, 727, 713, 702, 616. ^{1}H NMR (400 MHz, $CDCl_3$): δ (ppm) 8.78 (d, J = 8.3 Hz, 4H), 7.64 (dd, J = 8.5, 7.6 Hz, 4H), 7.35–7.49 (m, 8H), 7.20 (dd, J = 8.2, 7.6 Hz, 2H), 7.07 (dd, J = 7.8, 7.3 Hz, 2H), 6.86–6.77 (m, 4H), 6.63 (d, J = 7.6 Hz, 2H). ^{13}C NMR (100 MHz, $CDCl_3$): δ (ppm) 138.6, 137.7, 134.1, 133.5, 132.4, 132.2, 130.7, 129.8, 129.0, 128.7, 127.9, 127.6, 127.0 (two peaks), 126.7, 126.5 (two peaks), 126.4, 122.7 (two peaks). APCI-TOF MS: calcd. for $C_{46}H_{26}$ $[M]^+$: m/z = 506.20; found: 506.20.

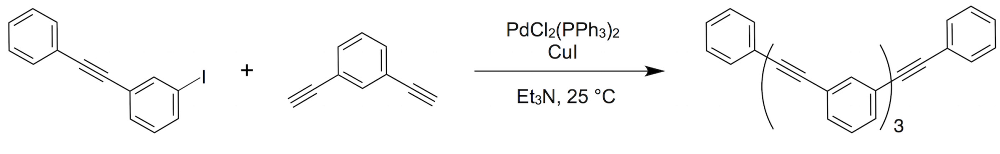

19

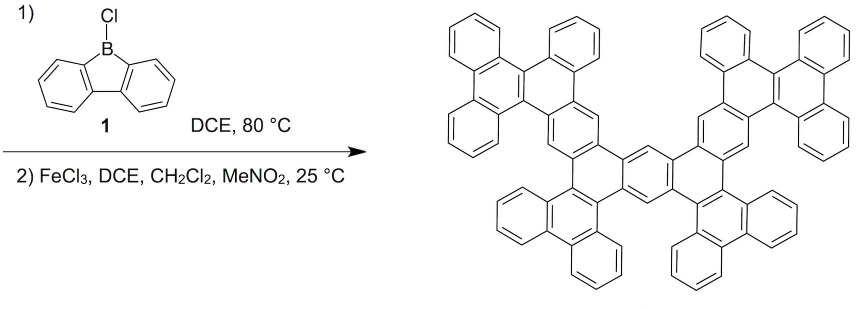

20

Synthesis of 1,3-bis((3-(phenylethynyl)phenyl)ethynyl)benzene (19). Under argon, 1,3-diethynylbenzene (153 μL, 1.2 mmol) was added to a mixture of dry Et_3N (5.0 mL), 1-iodo-3-(phenylethynyl)benzene[9] (700 mg, 2.3 mmol), $PdCl_2(PPh_3)_2$ (17 mg, 2.3 × 10^{-2} mmol), and CuI (5.2 mg, 2.3 × 10^{-2} mmol). After stirring for 18 h at 25 °C, the reaction mixture was poured into water and extracted with CH_2Cl_2. The organic layer was washed successively with saturated aqueous NH_4Cl and brine, dried over anhydrous $MgSO_4$, and evaporated to dryness under reduced pressure. The obtained residue was subjected to column chromatography on SiO_2 (hexane), which allowed the isolation of **19** as a colorless powder (253 mg, 0.529 mmol) in 46% yield: m.p.: 210 °C. FT-IR (KBr): ν (cm^{-1}) 3080, 3056, 3033, 2925, 1600, 1573, 1492, 1442, 1416, 1091, 1068, 1025, 966, 907, 795, 754, 684. ^{1}H NMR (400 MHz, $CDCl_3$): δ (ppm) 7.70–7.75 (m, 3H), 7.46–7.58 (m, 10H), 7.32–7.40 (m, 9H). ^{13}C NMR (100 MHz, $CDCl_3$): δ (ppm) 134.9, 134.8, 131.8, 131.7 (two peaks), 131.5, 128.7 (two peaks), 128.6, 128.5, 123.9, 123.6 (two peaks), 123.2, 90.2, 89.4, 89.2, 88.7. APCI-TOF MS: calcd. for $C_{38}H_{22}$ $[M]^+$: m/z = 478.1716; found: 478.1722.

Synthesis of 20. Under argon, a dry DCE solution (2.0 mL) of a mixture of **1** (97 mg, 0.49 mmol) and **19** (53 mg, 1.1 × 10^{-1} mmol) was stirred for 72 h at 80 °C and then allowed to cool to 25 °C. Dry CH_2Cl_2 (100 mL) and a dry $MeNO_2$ solution (1.0 mL) of $FeCl_3$ (717 mg, 4.4 mmol) were added successively to the reaction mixture under argon bubbling through a glass capillary. After stirring for 2 h at 25 °C, the resulting mixture was poured into MeOH (200 mL). The brown precipitate thus formed was collected by filtration, dissolved in $CHCl_3$, passed through a plug of silica gel, and evaporated to dryness. The obtained residue was subjected to SEC with $CHCl_3$ as the eluent, which allowed the isolation of **20** as orange crystals (55 mg, 5.1 × 10^{-2} mmol) in 46% yield: m.p.: >440 °C. FT-IR (KBr): ν (cm^{-1}) 3064, 3028, 1603, 1487, 1447, 1429, 1368, 1229, 1163, 1052, 926, 907, 872, 795, 760, 730, 661, 613. ^{1}H NMR (500 MHz, ODCB-d_4): δ (ppm) 10.51 (s, 1H), 10.42 (s, 2H), 9.58 (s, 3H), 9.44 (d, J = 7.7 Hz, 2H), 8.84–9.00 (m, 6H), 8.76 (d, J = 7.7 Hz, 2H), 8.63–8.70 (m, 2H), 8.60 (m,

8H), 8.11 (m, 2H), 7.75–7.83 (m, 7H), 7.52–7.71 (m, 10H), 5.05 (s, 1H). CP/MAS ^{13}C NMR (201 MHz): δ (ppm) 133–114. FT-ICR MS: calcd. for $C_{86}H_{46}$ $[M]^+$: m/z = 1078.3594; found: 1078.3594. ^{1}H NMR and CP/MAS ^{13}C NMR spectra, as well as the FT-ICR MS spectrum of **20** are shown in Supplementary Figs. 12, 13 and 14, respectively.

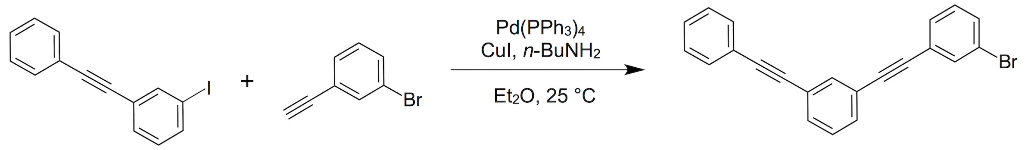

23

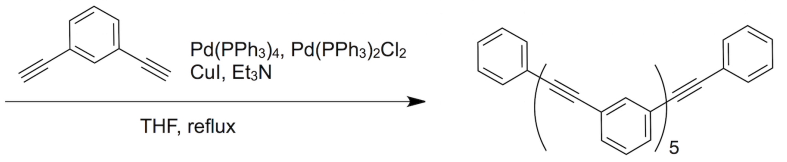

21

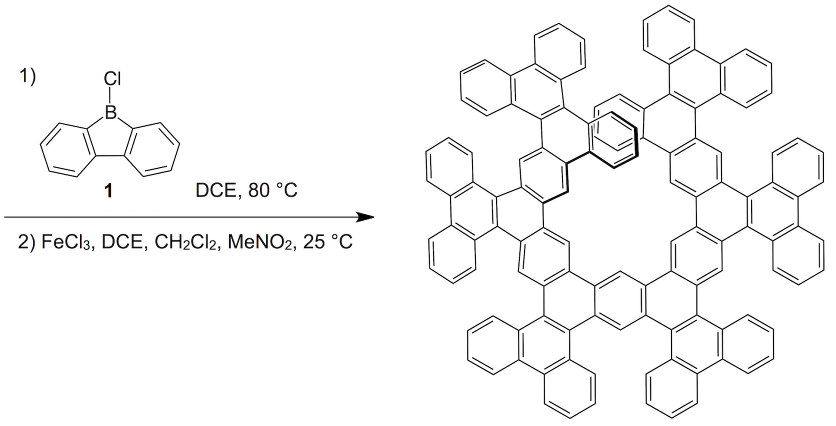

22

Synthesis of 1-bromo-3-((3-(phenylethynyl)phenyl)ethynyl)benzene (23): Under argon, 1-bromo-3-ethynylbenzene (650 mg, 3.6 mmol), CuI (70 mg, 0.36 mmol), and n-$BuNH_2$ (290 mg, 4.0 mmol) were successively added to a dry Et_2O suspension (82 mL) of 1-iodo-3-(phenylethynyl)benzene[9] (1.00 g, 3.3 mmol) and $Pd(PPh_3)_4$ (196 mg, 0.17 mmol). After stirring for 6 h at 25 °C, the reaction mixture was poured into a saturated aqueous solution of NH_4Cl and extracted with EtOAc. The organic layer was washed successively with water and brine, dried over anhydrous $MgSO_4$, and then evaporated to dryness under reduced pressure. The obtained residue was subjected to column chromatography on SiO_2 (hexane), which allowed the isolation of **23** as a white powder (1.0 g, 2.8 mmol) in 85% yield: FT-IR (KBr): ν (cm^{-1}): 2920, 1593, 1566, 1552, 1491, 1479, 1440, 1408, 1069, 906, 891, 787, 756, 681. ^{1}H NMR (500 MHz, $CDCl_3$): δ (ppm) 7.69 (d, J = 9.0 Hz, 2H), 7.54–7.44 (m, 6H), 7.36–7.32 (m, 4H), 7.22 (t, J = 8.0 Hz, 1H). ^{13}C NMR (125.7 MHz, $CDCl_3$): δ (ppm) 134.65, 134.35, 131.66, 131.63, 131.56, 131.29, 130.18, 129.81, 128.53, 128.49, 128.39, 125.03, 123.74,

123.09, 122.95, 122.20, 90.12, 89.81, 88.40, 88.34. APCI-TOF MS: calcd. for $C_{22}H_{13}Br$ $[M]^+$: *m/z* = 356.02; found: 356.02.

1,3-bis((3-((3-(phenylethynyl)phenyl)ethynyl)phenyl)ethynyl)benzene (21). Under argon, 1,3-diethynylbenzene (0.26 g, 2.06 mmol) was added dropwise to a dry THF suspension of **23** (1.79 g, 5.0 mmol), dry Et_3N (16 mL), $Pd(PPh_3)_2Cl_2$ (180 mg, 0.21 mmol), $Pd(PPh_3)_4$ (240 mg, 0.21 mmol), and CuI (40 mg, 0.21 mmol). The resulting mixture was stirred for 24 h under reflux and then allowed to cool to 25 °C. The reaction mixture was poured into water and extracted with $CHCl_3$. The organic layer was washed successively with water and brine, dried over anhydrous $MgSO_4$, and then evaporated to dryness under reduced pressure. The obtained residue was subjected to column chromatography on SiO_2 ($CHCl_3$/hexane: v/v = 1/2), which allowed the isolation of **21** as a white powder (860 mg, 1.3 mmol) in 60% yield: FT-IR (KBr): ν (cm^{-1}): 2358, 1644, 1598, 1489, 1145, 1090, 983, 792, 752, 681, 630. ^1H NMR (500 MHz, $CDCl_3$): δ (ppm) 7.73 (br, 5H), 7.54–7.47 (m, 14H), 7.38–7.34 (m, 11H). ^{13}C NMR (125.7 MHz, $CDCl_3$): δ (ppm) 135.42, 134.69, 134.65, 132.34, 131.66, 131.50, 131.31, 129.95, 128.57, 128.52, 128.47, 128.39, 128.00, 123.71, 123.46, 123.35, 122.99, 90.06, 89.22, 89.14, 89.06, 88.47. APCI-TOF MS: calcd. for $C_{54}H_{30}$ $[M]^+$: *m/z* = 678.23; found: 678.23.

Synthesis of 22. Under argon, a dry DCE solution (2.0 mL) of a mixture of **1** (97 mg, 0.49 mmol) and **21** (50 mg, 7.4×10^{-2} mmol) was stirred for 72 h at 80 °C and then allowed to cool to 25 °C. Dry CH_2Cl_2 (100 mL) and a dry $MeNO_2$ solution (1.0 mL) of $FeCl_3$ (717 mg, 4.4 mmol) were successively added to the reaction mixture under argon bubbling through a glass capillary. After stirring for 2 h at 25 °C, the resulting mixture was poured into MeOH (200 mL). The brown precipitate thus formed was collected by filtration, dissolved in $CHCl_3$, passed through a plug of silica gel, and evaporated to dryness. The obtained residue was subjected to SEC with $CHCl_3$ as the eluent, which allowed the isolation of **22** as an orange amorphous powder (35 mg, 2.2×10^{-2} mmol) in 30% yield: m.p.: >440 °C. FT-IR (KBr): ν (cm^{-1}) 3065, 3027, 2950, 2924, 1731, 1715, 1603, 1486, 1444, 1429, 1370, 1235, 1167, 1051, 1034, 948, 922, 870, 795, 760, 730, 616. ^1H NMR (500 MHz, $CDCl_3$): δ (ppm) 8.80 (br), 7.65 (br). CP/MAS ^{13}C NMR (201 MHz): δ (ppm) 133–114. FT-ICR MS: calcd. for $C_{126}H_{66}$ $[M]^+$: *m/z* = 1578.5159; found: 1578.5147. ^1H NMR and CP/MAS ^{13}C NMR spectra, as well as the FT-ICR MS spectrum of **22** are shown in Supplementary Figures 15, 16 and 17, respectively.

Single-crystal X-ray Diffraction Analysis. Crystallographic data are summarized in Supplementary Tables 1 and 2. Single crystalline pale-yellow blocks of **3a** and colorless blocks of **5** were obtained from hexane, while orange blocks of **9** were obtained from DCE. Orange blocks of **12** were obtained from toluene, colorless blocks of **14** were obtained from benzene, and orange blocks of **20** were obtained from CS_2/*m*-xylene.

For of **3a**, **5**, **9**, **12** and **14**, single crystals were coated with immersion oil (type B: Code 1248, Cargille Laboratories, Inc.) and mounted on a micromount. Diffraction data were collected at 90 K under a cold nitrogen gas stream on a Bruker APEX2 platform-CCD X-ray diffractometer system, using graphite-monochromated Mo-Kα radiation (λ = 0.71073 Å). Intensity data were collected by an ω-scan with 0.5° oscillations for each frame. Bragg spots were integrated using the ApexII program package[11], and the empirical absorption correction (multi-scan) was applied using the SADABS program[12]. Structures were solved by direct methods (SIR97)[13] and refined by full-matrix least squares (SHELXL97)[14]. Anisotropic temperature factors were applied to all non-hydrogen atoms. Hydrogen atoms were placed at calculated positions and refined applying riding models. For **12** and **14**, all the restraints were applied for solvents that are highly disordered in the voids. Because of the flexible and weakly binding nature of these solvents, geometrical restraints (DFIX) and atomic displacement parameter restraints (RIGU and ISOR) were used for the least-square refinement.

For **20**, a single crystal was coated with oil base cryoprotectant (Parabar 10312, Hampton Research Corp.) and mounted on a MicroLoops™. Diffraction data were collected at 93 K under a cold nitrogen gas stream on a Rigaku XtaLAB Pro MM007HF X-ray diffractometer system, using multi-layer mirror monochromated Cu-Kα radiation (λ = 1.54187 Å). Intensity data were collected by an ω-scan with 0.5° oscillations for each frame. Bragg spots were integrated using the CrystalClear program package[15], and the empirical absorption correction (multi-scan) was applied using the REQAB program[16]. Structures were solved by direct methods (SHELXT Version 2014/4)[17] and refined by full-matrix least squares (SHELXL Version 2014/7)[14]. Anisotropic temperature factors were applied to all non-hydrogen atoms. Hydrogen atoms were placed at calculated positions and refined applying riding models. The refinement was unstable because the crystal seems to have a pseudo-centrosymmetric structure. Therefore, the damping factor was applied using the DAMP command.

Supplementary References

1. Wrackmeyer, B., Tok, O. L., Milius, W., Bhatti, M. H. & Ali, S. Hydroboration of bis(trimethylsilyl)ethyne. New aspects of hydroboration. *Z. Naturforsch.* **58b**, 133–138 (2002).
2. Biswas, S., Oppel, I. M., Bettinger, H. F. Synthesis and structural characterization of 9-azido-9-borafluorene: monomer and cyclotrimer of a borole azide. *Inorg. Chem.* **49**, 4499–4506 (2010).
3. Mio, M. J. *et al*. One-pot synthesis of symmetrical and unsymmetrical bisarylethynes by a modification of the Sonogashira coupling reaction. *Org. Lett.* **4**, 3199−3202 (2002).
4. G. Bertrand *et al*., An improved protocol for the synthesis of $[(\eta^4\text{-}C_4R_4)Co(\eta^5\text{-}C_5H_5)]$ complexes. *Organometallics* **31**, 126−132 (2012).
5. Li, T., Qu, X., Xie, G., Mao, J. $[Cu(acac)_2]\bullet H_2O$-catalyzed Sonogashira-type couplings of aryl halides and terminal alkynes. *Chem. Asian J.* **6**, 1325–1330 (2011).
6. Yamaguchi, Y. *et al*., Rigid molecular architectures that comprise a 1,3,5-trisubstituted benzene core and three oligoaryleneethynylene arms: light-emitting characteristics and π conjugation between the arms. *J. Am. Chem. Soc.* **128**, 4504–4505 (2006).
7. Toyota, S. *et al*., Chemistry of anthracene–acetylene oligomers. XIV. Convenient synthesis of anthrylethynes by double elimination reaction from aldehydes and sulfones. *Bull. Chem. Soc. Jpn.* **82**, 1287–1291 (2009).
8. King, B. T. *et al*., Controlling the Scholl reaction. *J. Org. Chem.* **72**, 2279–2288 (2007).
9. Gagnon, E., Rochefort, A., Métivaud, V., Wuest, J. D. Hexaphenylbenzenes as potential acetylene sponges. *Org. Lett.* **12**, 380–383 (2010).
10. Nagata, T., Hirano, K., Satoh, T., Miura, M. Iridium-catalyzed annulative coupling of 2-arylbenzoyl chlorides with alkynes: selective formation of phenanthrene derivatives. *J. Org. Chem.* **79**, 8960−8967 (2014).
11. *SAINT,* version V7.60A, Bruker (2009), Bruker AXS Inc., Madison, Wisconsin, USA.
12. Krause, L., Herbst-Irmer, R., Sheldrick, G. M. & Stalke, D. *J. Appl. Crystallogr.* **48**, 3–10 (2015).
13. Altomare, A. M., Burla, C., Camalli, M., Cascarano, G. L., Giacovazzo, C., Guagliardi, A., Moliterni, A. G. G., Polidori, G. & Spagna, R. SIR97: a new tool for crystal structure determination and refinement. *J. Appl. Cryst.* **32**, 115–119 (1999).
14. Sheldrick, G. M. A short history of SHELX. *Acta Crystallogr. Sect. A* **64**, 112–122 (2008).
15. CrystalClear: Data collection and processing software, Rigaku Corporation (1998–2015), Tokyo, Japan.
16. *REQAB*, Molecular Structure Corporation (2008), The Woodlands, Texas, USA.
17. Sheldrick, G. M. SHELXT: Integrating space group determination and structure solution. *Acta Crystallogr. Sect. A* **70**, C1437 (2014).
